# Examining the Toxicity of α-Synuclein in Neurodegenerative Disorders

**DOI:** 10.3390/life11111126

**Published:** 2021-10-22

**Authors:** Frank Y. Shan, Kar-Ming Fung, Tarek Zieneldien, Janice Kim, Chuanhai Cao, Jason H. Huang

**Affiliations:** 1 Department of Anatomic Pathology, Baylor Scott & White Medical Center, College of Medicine, Texas A&M University, Temple, TX 76508, USA; 2 Department of Pathology, University of Oklahoma Medical Center, University of Oklahoma, Norman, OK 73019, USA; karming-fung@ouhsc.edu; 3 Department of Pharmaceutical Sciences, Taneja College of Pharmacy, University of South Florida, Tampa, FL 33620, USA; janicekim1@usf.edu (J.K.); ccao@usf.edu (C.C.); 4 Department of Neurosurgery, Baylor Scott & White Medical Center, College of Medicine, Texas A&M University, Temple, TX 76508, USA; Jason.huang@BSWHealth.org

**Keywords:** Parkinson’s disease, Lewy body, dopamine, substantia nigra, synucleinopathy, post-translational modifications

## Abstract

**Simple Summary:**

Neurodegenerative disorders are complex disorders that display a variety of clinical manifestations. The second-most common neurodegenerative disorder is Parkinson’s disease, and the leading pathological protein of the disorder is considered to be α-synuclein. Nonetheless, α-synuclein accumulation also seems to result in multiple system atrophy and dementia with Lewy bodies. In order to obtain a more proficient understanding in the pathological progression of these synucleinopathies, it is crucial to observe the post-translational modifications of α-synuclein and the conformations of α-synuclein, as well as its role in the dysfunction of cellular pathways.

**Abstract:**

α-synuclein is considered the main pathological protein in a variety of neurodegenerative disorders, such as Parkinson’s disease, multiple system atrophy, and dementia with Lewy bodies. As of now, numerous studies have been aimed at examining the post-translational modifications of α-synuclein to determine their effects on α-synuclein aggregation, propagation, and oligomerization, as well as the potential cellular pathway dysfunctions caused by α-synuclein, to determine the role of the protein in disease progression. Furthermore, α-synuclein also appears to contribute to the fibrilization of tau and amyloid beta, which are crucial proteins in Alzheimer’s disease, advocating for α-synuclein’s preeminent role in neurodegeneration. Due to this, investigating the mechanisms of toxicity of α-synuclein in neurodegeneration may lead to a more proficient understanding of the timeline progression in neurodegenerative synucleinopathies and could thereby lead to the development of potent targeted therapies.

## 1. Introduction 

**Figure 1:**
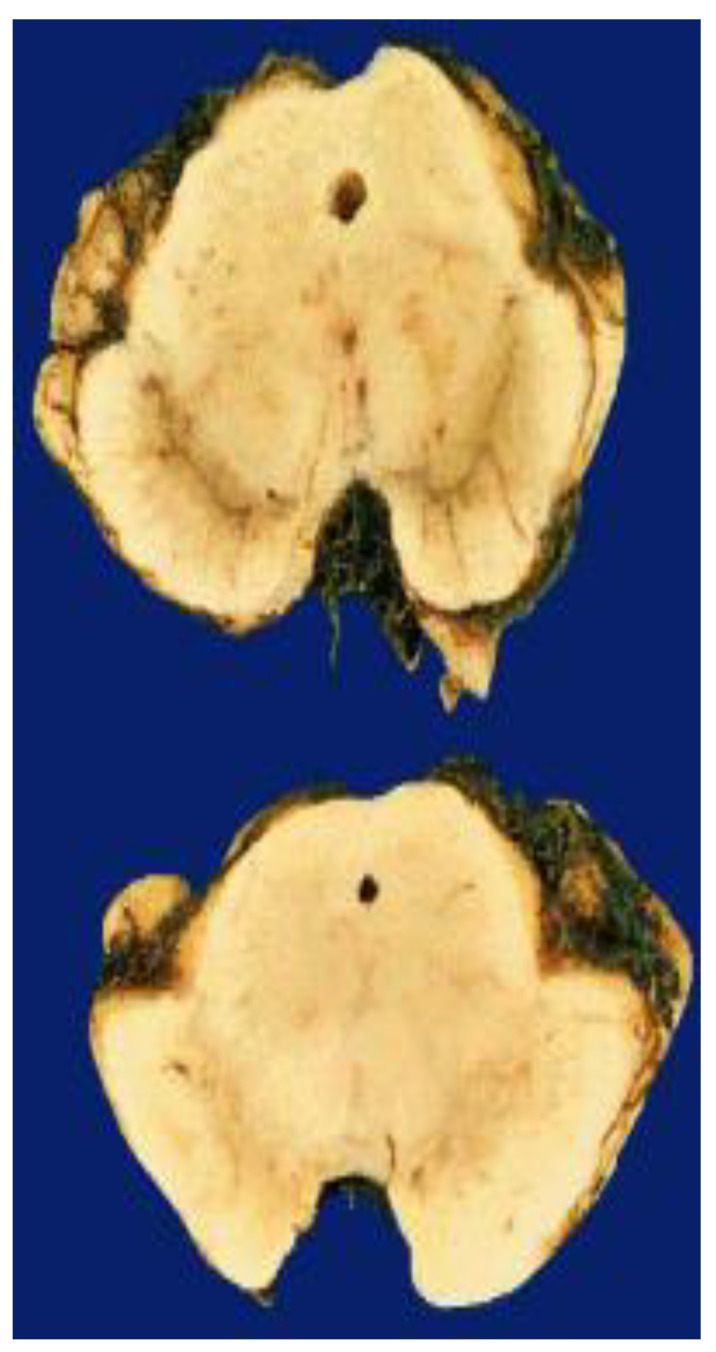
Gross picture of a midbrain section from a PD patient (**lower**) along with a normal control brain (**upper**), showing the PD patient’s substantia nigra with a loss of pigmented dopamine neurons.

Neurodegenerative disorders are heterogenous perplexing assemblages with disparate etiology and intermittently coinciding clinical manifestations [1]. Moreover, Parkinson’s disease (PD) is the second-most prevalent neurodegenerative disorder, causing over 6 million cases and 100,000 deaths at a global level annually [2]. In terms of pathology, PD is distinguished by an advancing reduction of dopaminergic neurons in the substantia nigra pars compacta (Figure 1) [3]. This leads to a striatal diminishing of dopamine in a location of the brain that is accountable for regulating fine motor control, eventually resulting in the tremors, bradykinesia, and rigidity often visualized in PD patients [4]. 

Over a century ago, Alois Alzheimer, Friedrich Lewy, and Oskar Fischer all made significant contributions to the descriptions of PD and AD pathological characteristics, which included neuritic plaques, Lewy neurites, and Lewy bodies, among others [5]. This is paramount when considering α-synuclein was determined to constitute Lewy neurites and Lewy bodies in the 1990s [6]. Even more, in 1997, an SNCA missense mutation, which is the gene for α-synuclein, was considered a familial PD causative factor [7]. Nevertheless, α-synuclein additionally accrues in differing synucleinopathies, such as multiple system atrophy (MSA), Lewy body dementia, and numerous lysosomal storage disorders [4,8]. 

**Figure 2:**
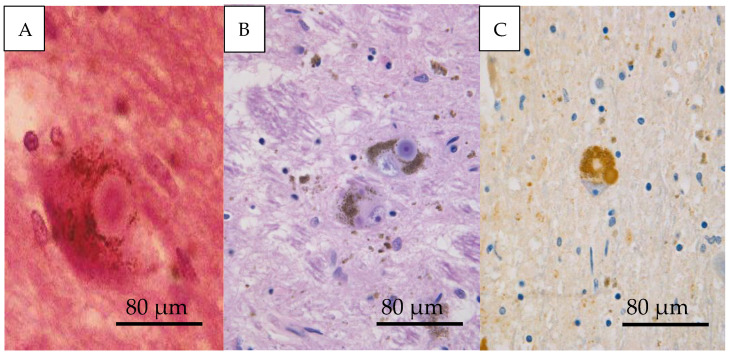
Microscopic pictures of a Lewy body. (**A**) Cytoplasmic inclusion body in pigmented dopamine neurons in the SN. (**B**) Lewy bodies in two neurons. (**C**) Immunohistochemical stain against α-synuclein demonstrates a positive stain for the Lewy body.

The accumulation of α-synuclein has also been witnessed in sporadic PD and proceeds towards the formation of Lewy bodies (Figure 2). Furthermore, α-synuclein point mutations, genomic duplications, and triplications in the locus of α-synuclein have been implicated in the dominant autosomal types of familial PD [9,10]. Similarly, α-synuclein has been determined to undergo a plethora of post-translational modifications that affect its function and structure and could potentially be associated with oligomeric or aggregated α-synuclein [11]. As such, this review aims to examine the functions, the post-translational modifications, the conformations, and the pathways implicated in the toxicity of α-synuclein. 

## 2. Properties, Functions, and History of α-Synuclein

Synucleins include various groups of soluble proteins found in vertebrates, and synucleins gained prominence when mutated α-synuclein, which is coded by SNCA, was commonly found in numerous families that had the autosomal dominant pattern of PD [7]. The name “synuclein” derives from the fact that it is expressed at the nuclear envelope and was first located with synapses [12]. Although α-synuclein is relatively abundant in the brain within as much as 1% of all cytosolic brain cell proteins, its specific function remains relatively unknown [13]. 

The first evidence of α-synuclein’s presynaptic role derived from the utilization of an antibody to cholinergic vesicles of the Torpedo electric organ [14]. Nonetheless, due to α-synuclein’s small size of 140 amino acid residues, it was suggested that cytoplasmic or nuclear proteins had an impact on α-synuclein’s effect on the nucleus [15]. When visualizing neural degeneration, the aggregation of synuclein into a β-sheet results from misfolding, which advocates for a prion model of propagation [16,17,18].

Even though the function of α-synuclein is not completely understood, it has been suggested that it is involved in modulating neurotransmitters due to their elevated presynaptic concentrations [8]. Often, this manifests in hindered synaptic vesicle mobility, which thereby reduces the release of neurotransmitters and the recycling of synaptic vesicles [19]. On the other hand, a differing perspective is that the binding of vesicle-associated membrane protein 2 (VAMP2) to α-synuclein contributes to SNARE complex stability [20]. VAMP2 and α-synuclein binding is crucial for the α-synuclein-mediated attenuation of the recycling of synaptic vesicles [21]. Similarly, α-synuclein was found to inhibit the synthesis of dopamine by serving as a tyrosine hydroxylase inhibitor [22]. This is critical in the case of PD, since dopaminergic neuronal loss in the substantia nigra is a significant manifestation of the disease [4]. 

Consequently, synuclein was found to be a phospholipase D2 (PLD2) inhibitor after purification, which distinguished a particular protein function via an experimental method [23]. The phosphatidylcholine headgroup is subsequently cleaved by phospholipase D (PLD), which thereby liberates phosphatidic acid and choline, which have been involved in regulated exocytosis via trafficking in the membrane [24]. Nevertheless, this function has not been entirely proven by consequential studies [25]. This yields a function with slight biological applicability, because the biochemical activity suggested by purified synuclein as a PLD inhibitor could be useful but has not yet been elucidated in further studies. 

The ability of α-synuclein to be secreted has also been established in a plethora of cell models in vitro, in human body fluids, and in a mouse brain in vivo [26,27,28,29]. El-Agnaf et al. demonstrated that α-synuclein species could be detected in CSF and human plasma and also secreted into mediums of cultured neurons [27,28]. Subsequently, another study elucidated that oligomeric and monomeric α-synuclein were demonstrated to be secreted from primary cortical neurons and differentiated neuroblastoma human cells [30]. Utilizing a comparable model, Sung et al. showed that α-synuclein secreted from SK-N-BE cells minimizes the cell viability and can be cleaved by the matrix metalloproteases [31]. Although the pathway of secretion is currently not defined, it appears to involve exosome externalization [26]. Exosomes are vesicles that have the ability to interact with recipient cells in a plethora of ways, such as attachment, endocytosis, receptor–ligand binding, or fusion with the plasma membrane [32,33,34]. Consequently, the extracellular degradation of exosomal membranes by lipases or proteases could thereby allow protein release into the extracellular matrix from the exosomal lumen [35,36]. In general, α-synuclein exportation from the cell via exosomal pathways postulates a prevailing pathway for possibly toxic protein delivery in the extracellular space, leading to the spread of pathogenic effects in healthy neighboring cells [26].

An autosomal dominant type of PD that caused the usual bradykinesia, rigidity, impaired posture, and tremors was also discovered to be caused by an α-synuclein point mutation [37]. Some point mutations include A30P, G51D, E46K, A53T, and A53E [9,10]. In this case, the pathology revealed inclusions of Lewy bodies typical for PD [38]. Although α-synuclein point mutations account for a small percentage of PD, the dystrophic neurites and Lewy bodies seen in the most common type of PD, idiopathic PD, greatly imply α-synuclein [8,37]. This is further demonstrated with a plethora of monoclonal antibodies recognizing α-synuclein when they were formerly made against Lewy bodies [39]. This upholds the belief that, even though various proteins compile in PD inclusion, the α-synuclein protein preponderates [40].

## 3. Post-translational Modifications of α-Synuclein in PD

Although the definite role of α-synuclein is currently undetermined, the formation of α-synuclein oligomers, as well as aggregation, have been exhibited to go through an array of differing post-translational modifications, which are postulated to be vital in PD pathogenesis [11,13]. 

### 3.1. Phosphorylation 

Although numerous post-translational modifications are affiliated with α-synuclein, phosphorylation is the modification that is most researched. Consequently, S129 phosphorylation, as well as the truncation of α-synuclein, are determined to be pivotal to Lewy inclusion pathogenesis [41,42]. Furthermore, α-synuclein detected in the soluble nonfibrillar portions of PD patient tissue were found to be phosphorylated at S129 [43]. Due to this, α-synuclein post-translational modification was determined to potentially reform the disposition to transform into oligomers or even aggregate, thereby impacting PD inception or advancement [44]. Nevertheless, alternative residues can undergo post-translational modifications in α-synuclein, such as residues Y125, Y133, Y136, or S87 [44]. However, out of the discovered phosphorylated residues, S129 is extensively studied more than the others and also manifests as more pathologically relevant than the others in the case of PD [45,46]. Nonetheless, the specific outcome of S129 phosphorylation is currently undergoing further investigation. As of now, research has revealed alternative outcomes pointing to potential protection or toxicity based on the model utilized [47]. For example, some studies have revealed that S129 phosphorylation yields protection in rat and yeast models [48,49]. Moreover, a number of research studies have illustrated that phosphorylated S129 stimulates the creation of Lewy body inclusions and induces toxic effects [50]. Similarly, *Drosophila* models have revealed that phosphorylated S129 is associated with a more rapid loss of neurons when examined in comparison to wild-type (WT) or non-phosphorylated mutants, thereby leading to the phosphorylation of S129 to be associated with the pathology of PD [50,51]. 

Furthermore, α-synuclein also appears to be affected by phosphorylation-related post-translational modifications via the debilitated function of the mitochondria in PD [52]. In fact, various studies have deduced that phosphorylation events are essential for mitochondrial-targeting proteins [53,54]. However, subsequent research studies are necessitated in the field to further establish the influence of the phosphorylation of α-synuclein on debilitated mitochondrial functions and how they affect PD pathology. 

### 3.2. Nitration

When examining PD patient brains, their dopaminergic neurons have shown elevated oxidative stress, as revealed by optimized marker detections for DNA, lipid, and oxidized proteins [55]. In general, injuries of an oxidative nature take place after the brain’s antioxidant capacity has been overpowered and, hence, cannot handle the produced reactive oxygen species (ROS) [56]. To illustrate, a prevalent consequence of elevated oxidative stress unfolds with tyrosine residue nitration by peroxynitrite, which is a product of nitric oxide and oxygen reacting. In the Lewy bodies, similar to phosphorylation, distinct antibodies have been utilized to distinguish numerous types of 3-nitrotyrosine-modified α-synuclein [57]. Subsequent investigations have revealed tyrosine nitrations at four other positions, which include residues Y39, Y125, Y133, and Y136 [58]. 

Research on the nitration of α-synuclein has elucidated numerous clues on the aggregation of the protein. In general, tyrosine nitration has been shown to affect α-synuclein by promoting dimer and oligomer formations due to the crosslinking of dityrosine [44,59]. However, research has also shown that nitrated α-synuclein can prevent the formation of fibrils, and this was identified when unmodified α-synuclein was incubated with the nitrated version of α-synuclein, and this coincubation yielded nitrated α-synuclein that was embodied into unmodified α-synuclein fibrils [60]. Furthermore, nitrated Y39 has demonstrated the ability to impede α-synuclein lipid binding [61]. When considering both of these potential effects, it could be indicated that the nitration of α-synuclein could act analogous to mutated A30P, which has been shown to cause the early onset of PD [62].

Numerous elements are involved in the elevation of oxidative stress in dopaminergic neurons [63]. Consequently, nigral neurons have been found to be more prone towards oxidative stress and the production of ROS [63,64]. Even more, senility has been associated with declined mitochondrial function and antioxidative defense while also increasing the prospect of oxidative injuries, which encompasses post-translational nitration of the proteins [65,66]. As such, this brings into question whether the nitration of α-synuclein is a byproduct of PD pathogenesis or if it is a critical post-translational modification that occurs antecedently to the disease and is toxic. Nevertheless, the nitration of tyrosine has the potential to mimic certain aspects of mutated A30P, which could aid in the progression or initiation of PD [67]. 

### 3.3. Dopamine Modification 

In the substantia nigra, dopamine is usually found and concentrated within dopaminergic neurons, where it is located in synaptic vesicles [68]. Dopamine has the ability to auto-oxidize at a neutral pH into the toxic dopamine-quinone, because it is immensely reactive [69]. Similarly, it can also produce hydrogen peroxide, as well as superoxide radicals [63]. The production of the toxic species, along with the declining levels of antioxidants like glutathione, which is witnessed in PD and aging, has the potential to induce oxidative stress, which can lead to adverse effects in cells [70,71]. Furthermore, elevated cytoplasmic dopamine has also been seen in PD [72]. Typically, dopamine is usually found in synaptic vesicles, which averts the formation of dopamine-quinone because of its low pH [73]. Nonetheless, earlier research has associated the elevated permeability of synaptic vesicles to mutated A30P and A53T and even oligomeric α-synuclein, possibly associating elevated dopamine levels of the cytoplasm in PD patients with α-synuclein toxic activity [74,75]. To illustrate, mutated A53T α-synuclein has been shown to cause a decline in the vesicular monoamine transporter 2 levels, which is a protein that is involved in the vesicular uptake of dopamine [72]. The decline of vesicular monoamine transporter 2 may similarly lead to the surge of dopamine found in the cytoplasm [76]. Surprisingly, a heightened tyrosine hydroxylase expression has demonstrated the ability to hinder the aggregation of α-synuclein, as well as cause elevated toxic effects in SH-SY5Y cell lines [77,78]. This associates elevated levels of dopamine with changes in the aggregation of α-synuclein, because tyrosine hydroxylase is involved in the generation of dopamine as the rate-limiting enzyme [79].

Nevertheless, the gravity of elevated dopamine levels in the cytoplasm is not limited to the adverse products generated by oxidized dopamine [68]. In fact, it has been hypothesized that dopamine has the potential to modify α-synuclein, thereby altering its disposition to aggregate [80,81]. Early studies conducted by Conway et al. demonstrated that dopamine-quinone stabilizes α-synuclein protofibrils in a kinetic manner while also preventing the aggregation of α-synuclein into considerable fibrils [5]. These research findings were subsequently backed by Li et al., who similarly identified that α-synuclein modified by dopamine had the ability to de-aggregate fibrils, potentially by debilitating the intermolecular forces encountered in the fibrils, generating species of oligomers that were relatively stable [82]. Consequently, further research elucidated that dopamine modification was usually covalent, since dopamine modification generates oligomers that were insusceptible to sodium dodecyl sulfate (SDS) [83]. Nonetheless, a counterpoint raised by other studies suggested that this interaction was transient and transpired within the C-terminal domain in the Y_125_EMPS_129_ region, but the research that ensued showed that this region was not crucial for dopamine modification, since truncation research elucidated the inhibited formation of fibrils [84]. Currently, there is increasing evidence that dopamine modification eventuates as a crosslinking of dopamine in a stoichiometric manner amidst polymers of dopamine at residues of tyrosine and lysine and α-synuclein [85]. This reaction leads to a steady generation of α-synuclein-dopamine trimers that have been examined via the small angle X-ray scattering technique and has shown a “worm-like” structure devoid of the usual beta-sheet structures typically affiliated with the fibrils of α-synuclein [86]. Furthermore, the α-synuclein-dopamine trimers also lacked certain structural aspects, such as helical elements, implying that oligomers stabilized by dopamine could differ from those examined in earlier research [86]. 

Nonetheless, it is challenging to deduce whether the dopamine modifications of α-synuclein are causative or if it is promoted by PD pathogenesis [87]. In general, it seems that the dopamine modification of α-synuclein materializes only in circumstances of elevated dopamine levels in the cytoplasm or oxidative stress [88,89]. 

### 3.4. Sumoylation, O-GlcNAcylation, Ubiquitination, and Truncation

In essence, α-synuclein usually experiences a vast array of post-translational modification and in itself is a protein with substantially unidentified functions (Table 1) [13,44]. Although phosphorylation, nitration, and dopamine modifications are typically correlated with PD, they are not the only post-translational modifications that ensue [44]. However, out of the most commonly correlated post-translational modifications, there appears to be an evident association of the modifications and oligomerization that can be deduced [90]. Similar to inherited α-synuclein mutations, phosphorylation, nitration, and dopamine modifications seem to stabilize and sustain the protofibril state of α-synuclein while simultaneously disaggregating the extensive insoluble fibrils encountered in Lewy body pathological conditions [74,91]. Nevertheless, as previously discussed, there is contradicting and conflicting research in the field, so it is currently unclear if oligomeric species display elevated toxicity and are liable for PD progression and pathogenesis [91,92].

As of now, one of the main challenges remaining is deducing what role the other post-translational modifications have in oxidative stress and mitochondrial dysfunction in PD pathology. Phosphorylation, nitration, and dopamine modifications have all been associated with mitochondrial dysfunction, and accumulating data advocates that these modifications have a direct role [11,44]. Furthermore, the current research also suggests a bidirectional interaction between protein aggregation and dysfunction of the mitochondria [93]. To illustrate, inhibiting mitochondrial complex I has been shown to cause the oligomerization and accumulation of α-synuclein [44]. Due to this, data associating α-synuclein with dysfunction of the mitochondria might be crucial to obtaining insight on the commencement of events that lead to the onset of PD. 

Consequently, O-GlcNAcylation and sumoylation have also been examined as post-translational modifications [94,95]. O-GlcNAcylation has been identified at T72, T75, T81, and S87 and has been found to inhibit the aggregation of α-synuclein [94]. In general, O-GlcNAcylation occurs when N-acetylglucosamine is transferred to the threonine and serine residues of proteins via O-GlcNAc transferase and subsequently excised by O-GlcNAcase [94]. On the other hand, sumoylations have been recognized at K96 and K102 and have been determined to elevate aggregated levels of α-synuclein via the promotion of PIAS2 and the impairment of α-synuclein degradation via the defected ubiquitination of the protein [95,96]. However, there have been contrasting reports that revealed that sumoylation actually inhibits the aggregation of α-synuclein [95]. As for ubiquitination, K10, K12, K21, K23, K34, K43, and K96 have been identified [11]. In terms of ubiquitination, in vitro and in vivo studies have elucidated that α-synuclein ubiquitination boosts the production of inclusions via the seven in absentia homolog (SIAH) protein [11,97]. Further experiments also revealed that the in vitro ubiquitination of α-synuclein boosts the production of an α-synuclein form with a larger molecular weight, and electron microscopy further showed that ubiquitinated α-synuclein produced more aggregated forms via SIAH [97,98]. 

Other than full-length α-synuclein, there are minute amounts of numerous truncated α-synuclein species with molecular masses of approximately 10-15 kDa in the Lewy Bodies [99,100]. As for truncation, K58, V74, K80, and K97 have been identified [101]. The enzymes neurosin, Matrix metalloproteinase 3, calpain I, and Cathepsin D have been determined as implicit in the truncation of α-synuclein [101,102]. Since the localization of α-synuclein is generally determined to be the presynaptic terminal, it could be a substrate for membrane-associated proteases like calpain I [102]. Mishizen-Eberz et al. showed that calpain I cleaves WT α-synuclein after amino acid 57, as well as within the NAC region at amino acids 73, 74, and 83 [102]. The calpain-mediated processing of soluble α-synuclein was determined to inhibit fibrillization [102]. On the other hand, the processing of fibrillar α-synuclein appeared to stimulate aggregation [11]. Consequently, neurosin, which is a serine protease mainly expressed in the central nervous system (CNS), is presupposed to have a crucial role in α-synuclein’s degradation [103]. Neurosin cleaves α-synuclein subsequent to amino acids 80, which potentially inhibits polymerization, and 97, which has a more robust propensity to polymerize when compared to non-processed α-synuclein [101]. 

## 4. Conformations of α-Synuclein

The native conformations of α-synuclein include monomers and tetramers, with α-synuclein having the ability to transition between various differing conformations, ranging from monomers and tetramers to soluble oligomers and insoluble fibrils and aggregates [80,106]. The initial research showed that α-synuclein is typically found as a monomer in its native form, and the current evidence has demonstrated that α-synuclein may adopt a compact monomeric nature in its native state [107]. This compact monomeric form allows α-synuclein to protect the non-amyloid-beta component area from aggregating spontaneously [107]. Furthermore, α-synuclein has similarly been found to occupy a relatively stable monomeric form and a metastable form while still having the ability to occupy a tetramer conformation that is arbitrated by the repeating sequence of the KTKEGV segment [107,108]. Consequently, α-synuclein mutations such as E46K and A53T, which are commonly associated with PD, have been shown to elevate their monomeric form while reducing the tetrameric conformation, advocating for the unfolded monomer potentially being an element in the toxicity of α-synuclein [109,110]. As such, α-synuclein may have a plethora of native conformations based on the specific membrane interactions and locations within the cell. 

Fibrils and oligomers are considered to be toxic conformations of α-synuclein [111]. There is currently a great amount of research examining the factors that encourage the initiatory formation of α-synuclein oligomers, and various factors have been found to elevate the oligomeric α-synuclein levels, such as polyunsaturated fatty acids and the moderately acidic environment of the lysosomes and endosomes [8,112]. However, certain factors, such as saturated fatty acids, cause a decline in the oligomeric α-synuclein levels [113]. Nevertheless, in order to adopt a stable form, oligomeric α-synuclein must go through changes in their conformation that stabilize and consolidate oligomers that are resistant to proteinase-K, which generate inflated oxidative stress prior to fibril formation [114]. Consequently, α-synuclein demonstrates a high affinity to a wide variety of cellular membranes, thereby leading to the potential contribution of membrane lipid constituents to synuclein dysfunction on the surface of the membrane [115]. Furthermore, as previously mentioned, fatty acids drastically alter the conformation of α-synuclein. To further elucidate, saturated fatty acids have been shown to decrease the oligomeric levels of α-synuclein, while polyunsaturated fatty acids have been demonstrated to increase the α-synuclein oligomeric levels [112,113,116]. Even more, a low quantity of negatively charged lipids, lipid vesicles, and mildly acidic environments, such as those encountered in lysosomes and endosomes, all appear to stimulate oligomerization [117,118,119].

There have been studies aimed at investigating which form, oligomers or fibrils, is the most toxic conformation of α-synuclein [111,120,121]. Some studies indicated that the oligomeric form may exhibit a higher toxicity, since α-synuclein transgenic mice, as well as PD and dementia with Lewy bodies patients, demonstrated elevated levels of soluble, lipid-dependent oligomers of α-synuclein in the brain in comparison to the controls [113]. Furthermore, α-synuclein PD-associated A30P and A53T mutations further seem to expedite oligomerization, although not fibrillization [7,122]. Likewise, α-synuclein variant injections boost oligomerization, but not fibril formation, in the brains of rats, which has been shown to lead to a more drastic loss of dopaminergic neurons [121]. 

On the other hand, recent studies have elucidated that α-synuclein fibrils may be up to 1000 times more toxic than their precursors, with different human α-synuclein assembly injections into the substantia pars nigra compacta (SNC) of rats, having demonstrated that fibrils may induce and influence a more preeminent motor impairment, synaptic impairment, and loss of dopaminergic neurons than oligomers or ribbons [123,124]. As such, it is crucial to continue exploring the roles of the differing conformations of α-synuclein, such as fibrils, oligomers, and even ribbons, since it would aid in the discernment of α-synuclein toxicity [125,126,127].

α-synuclein has also been noted to have at least three varying strains that show differing properties, such as toxicity, ability to propagate, structural differentiations, and cross-seeding tau fibrillization [128,129]. Even more, when observing synucleinopathies in MSA and PD, there have also been noted differences in the α-synuclein strains [6,130]. To illustrate, the brain extracts of MSA patients, not PD patients, showed increased neural atrophy and functional loss once injected into the transgenic mice, implying that the α-synuclein strains obtained from MSA patients may exhibit a higher toxicity [17]. Consequently, MSA brain extracts from various patients also revealed differing α-synuclein transmission rates, propounding that, within MSA, there appears to be varying α-synuclein strains even in patient-to-patient scenarios [18]. Due to these reasons, the variability in α-synuclein strains may lead to differing conditions in patients, including the rate of pathological progression, age of onset, and severity of disease [8]. 

## 5. Pathways Implicated in α-Synuclein Toxicity

In general, α-synuclein manifests with various conformations and is relatively intrinsically disordered, encompassing amyloidogenic oligomeric forms [107]. Furthermore, α-synuclein also has three differing parts: a hydrophobic non-amyloid-beta component that has been recently crystallized, which supplements the oligomer formation, a carboxy-terminal that is intrinsically disordered, and an amino region that binds lipids [131]. Even though α-synuclein is typically located at the presynaptic terminal, the oligomeric forms and aggregates are usually found dispersed in the neurites and cell body, implying that α-synuclein has the potential to disturb the cellular function further away from the presynaptic terminals [8]. Consequently, a plethora of organelles are involved in the toxicity of α-synuclein, inclusive of Golgi, lysosomes, nuclei, autophagosomes, synaptic vesicles, mitochondria, and ER [8,132]. Even more, α-synuclein also disturbs the axonal transportation of organelles and the inter-organelle contacts [133,134].

α-synuclein’s presynaptic localization allows it to be affiliated with the synaptic vehicles, which thereby participate in the binding of membranes and causes a curvature of the membrane [135,136]. In general, α-synuclein functions in the regulation of the NSF attachment protein receptor (SNARE) soluble complex by encouraging the fusion of the synaptic vesicles at the presynaptic terminal via SNARE synaptobrevin-2/vesicle-associated membrane protein 2 (VAMP2) binding [136,137]. Furthermore, it also possibly functions in the regulation of the trafficking of synaptic vesicles [19,138]. Nonetheless, larger oligomeric forms of α-synuclein seem to be inclined towards VAMP2 binding, thereby disrupting the complex formation of SNARE and leading to the release of dopamine, as well as the motility of the synaptic vesicles [21]. Even more, elevated α-synuclein might also disturb the release of neurotransmitters via the reduced recycling of synaptic vesicles in the circulation and mobility [21]. Similarly, increased α-synuclein also has the potential to disturb the neurotransmission of dopamine, which has been witnessed in α-synuclein-deficient mice, since they revealed elevated levels of dopamine discharge from the nigrostriatal dopamine system, even though deletion of the α-synuclein protein should not affect the quantity of dopamine in the cytosol [139]. Furthermore, transgenic mice with an upregulation of human α-synuclein have demonstrated a loss of dopaminergic neurons, decreased release of dopamine, and modified distribution of the synaptic vesicles [140]. Likewise, the elevated expression of α-synuclein has also been correlated with defective activity of the dopamine transporter, as well as a decline in the reuptake of dopamine, thereby indicating that there are numerous possible processes in which α-synuclein has the potential to disrupt the dopamine levels [140,141,142,143]. 

Mitochondria, which are critical for the synthesis of ATP, the metabolism of lipids, the storage of calcium, and the survival of neurons, are potentially disrupted by α-synuclein [144]. In general, α-synuclein toxicity has been shown to disturb mitochondrial homeostasis, since A53T-mutated mice have shown elevated levels of mitophagy and mitochondrial DNA damage [144]. Furthermore, elevated levels of α-synuclein seem to boost dynamin-related protein 1 (DRP1)-independent mitochondrial fission in mouse models with α-synuclein upregulation, as well as in cell lines [145]. Subsequently, mice that lack α-synuclein appear to have prevented dopaminergic neuron degeneration caused by 1-methyl-4-phenyl-1,2,3,6-tetrahydropyridine (MPTP), potentially due to mitochondrial dysfunction by oligomeric α-synuclein occurring due to an elevated calcium absorption [146,147]. α-synuclein that has undergone post-translation modifications has also been noted to disrupt the functions of the mitochondria by impairing the mitochondrial importance of proteins [147]. Nevertheless, dysfunction of the mitochondria could also potentially be indirectly instigated by toxic α-synuclein due to reduced amounts of the PGC-1α factor, which mediates mitochondrial biogenesis via the regulation of numerous transcriptional factors, as well as nuclear receptors [148]. Inhibition of the MEF2C-PGC-1α transcriptional network of the mitochondria has been seen in PD models of pluripotent stem cells that were induced by dopaminergic α-synuclein [149]. The mechanism seems to be related to the elevation of myocyte enhancer factor 2 (MEF2) S-nitrosylation [149]. 

In regard to the endocytic pathway, α-synuclein seems to disturb the endoplasmic reticulum function of trafficking to the Golgi in yeast, as well as actuate endoplasmic reticulum stress [132,150]. Even more, it also has the potential to disrupt the initial stages of the secretory pathway, which is aided by the RAB1, RAB3A, and RAB8A GTPases, among others [151]. Elevated levels of α-synuclein also appear to disturb endosomal transports by interfering with the E3 ubiquitin ligase of RSP5 in yeast, as well as NEDD4, which is the homolog found in mammals [150]. Nevertheless, endosomal transport is improved by treatment with N-aryl benzimidazole, which also has shown neuronal protection in various animal models [152]. Subsequently, inflated levels of α-synuclein also appear to disturb dopamine transporter trafficking, as well as increase the calcium found in the cytoplasm, which thereby causes incitement of the noxious cascade of calmodulin-calcineurin [153]. This alludes that α-synuclein potentially disturbs the buffering of calcium in the endoplasmic reticulum [154]. Furthermore, α-synuclein has also been considered to adhere to the GRP78 endoplasmic reticulum chaperone and hinder the folding machinery of the endoplasmic reticulum [155]. 

Autophagy is a crucial process that degrades organelles once damaged and even aggregates proteins [156]. In this case, upregulated α-synuclein disturbs the endoplasmic reticulum to Golgi apparatus trafficking by potentially focusing on the transmembrane ATG9 protein, thereby reducing the production of omegasomes, which are a precursor for the generation of autophagosomes [157]. When observing autophagy that is mediated by chaperones, the A30P and A53T-mutated α-synuclein seem to adhere to the LAMP2A receptors in the lysosome more securely than WT α-synuclein, effectively averting the deterioration [158]. Subsequently, α-synuclein that contains dopamine modifications appears to obstruct autophagy mediated by chaperones, potentially assisting in PD discriminatory dopaminergic susceptibility [159]. When examining neuronal models that were incubated with pre-established fibrils of α-synuclein, autophagosomal generation occurred as typically expected but seemed to have abnormal autophagic cargo accumulation, as well as lysosomal fusion, possibly attributable to faulty axonal transportation of the autophagosomes [133]. Consequently, since productive autophagic degradation depends on the proper enzymatic function of the lysosome, the activity of the lysosome in differing enzymes, such as cathepsin B, hexosaminidase, and Gcase, seems to decline in α-synuclein PD-induced pluripotent stem cells in comparison to control-induced pluripotent stem cells, potentially due to faulty endoplasmic reticulum to Golgi apparatus trafficking [156,160,161]. 

Despite the fact that α-synuclein was initially localized at the nucleus, this determination has since been contested, possibly because of the antibody utilization against cleaved α-synuclein [8,12]. To elucidate, α-synuclein targeted in the nucleus has been suggested to be regulated by tripartite motif-containing 28 (TRIM28), which is a nuclear protein, and the inhibition of histone acetylation has also been seen [162,163]. Furthermore, the α-synuclein G51D, A30P, and A53T mutations that are affiliated with PD have also shown heightened nuclear localization in comparison to the WT α-synuclein [163,164]. Consequently, the modified activation of a plethora of transcription factors has similarly been noticed, inclusive of a reduced activation of PGC-1 alpha in α-synuclein-mutated A53T PD-induced pluripotent stem cells of a patient, specifically of transcription factor EB (TFEB), which regulates the autophagy–lysosomal pathway in rats with heightened α-synuclein expression via adeno-associated virus [165]. The heightened activation of nuclear factor activated T cells from cell lines with activated calcineurin with upregulated WT or mutated α-synuclein (A53T) was also seen in the brains of PD patients, as well as transgenic mice [153]. 

Previously, various inter-organelle contacts have emanated as locations of cellular homeostatic regulation, including the mitochondria-associated endoplasmic reticulum membrane. This is a subdomain of the endoplasmic reticulum manacled to the mitochondria by an assemblage of adaptor proteins, which act as critical locations for the biogenesis of autophagosomes, the homeostasis of calcium, the transport of phospholipids, and the fission of the mitochondria [166]. Recently, two conflicting studies have suspected that the mitochondria-associated endoplasmic reticulum membrane has a role in α-synuclein toxicity, with one study finding that the membrane elevates the contact locations, thereby leading to a heightened uptake of mitochondrial calcium from the endoplasmic reticulum during the upregulation of α-synuclein, and the other study recognized α-synuclein in membrane fractions and reported reduced amounts of the membrane contact sites in WT, A30P, and A53T-mutated α-synuclein [167,168]. Nonetheless, both studies reported the fragmentation of the mitochondria [169]. As such, additional research is required to account for the discrepancies, including neuronal experiments, which are critical for the apprehension of α-synuclein interactions with the mitochondria-associated endoplasmic reticulum membrane, along with the actions of the numerous inter-organelle contacts in relation to α-synuclein’s toxic effects [8]. 

Furthermore, the propagation capacity of α-synuclein is a crucial molecular mechanism that contributes to the advancement of PD. Even though the dopaminergic neurons of the substantia nigra appear to be notably vulnerable in PD, the observation of PD advancement elucidates that α-synuclein pathology is not limited to this region. Consequently, in 2003, Braak et al. postulated the hypothesis that the advancement of α-synuclein pathology pursues a particular caudo-rostral pattern through the CNS [170,171]. Subsequently, Braak et al. presupposed that the two PD starting points were the enteric nerves and the olfactory bulb, with damage occurring via the vagus nerve or the olfactory tract, respectively, to the other regions of the brain [170,171]. This theory divides PD into six stages, with each stage being distinguished by the development of α-synuclein inclusions in particular brain areas, such as magnocellular portions of reticular formation, substantia nigra, cortex, dorsal motor nucleus of the vagus nerve, locus coeruleus, and raphe nuclei [171,172]. The existence of these α-synuclein inclusions generates dysfunctionalities in the cells, which have been determined to be responsible for the development of clinical PD pathology and symptoms [170].

Numerous researchers have examined the cellular toxicity of α-synuclein at a steady state [8]. Nonetheless, organelles are exceptionally dynamic structures that go through fusion, fission, axonal transport, and maturation [173]. Even more, they are elaborately regulated by a plethora of signaling pathways that are arbitrated by electrical and calcium signaling, phosphorylation, and Rab GTPases [8]. Furthermore, in recent times, the fibrils of α-synuclein have been discovered to induce an impairment in the axonal transport of TrkB and RAB7-positive endosomes [133]. However, this effect was not observed in the transport of mitochondria or even synaptophysin, implying that α-synuclein does not induce irregularities in axonal transport [133]. In general, this could partly be attributed due to reduced levels of the axonal transport proteins seen in sporadic PD patients in comparison to the controls that were matched by age or even the reduced stability of microtubules and kinesin-dependent mobility, as seen in oligomeric α-synuclein cellular studies [174,175]. Defections in the transportation systems could also be arbitrated by tau interactions with α-synuclein, a protein that stabilizes and bolsters the assembly of microtubules [129]. Subsequently, exposure of the neurons to extracellular α-synuclein has also been found to disturb the actin waves along the axons, as well as the turnover of the actin protein, due to the inactivation of cofilin [176]. On the other hand, α-synuclein’s role in vesicle fusion and fission regulation, as well as the dynamics of maturation in neurons, is an area that warrants additional research contributions, since there is a lack of data [8]. Thus, expanding our knowledge in these processes could potentially further elucidate α-synuclein’s neurodegenerative actions. 

There have been various studies that have depicted numerous cellular dysfunction pathways when modeling the toxicity of α-synuclein [127,177]. In general, it is plausible that the differing pathways could be afflicted in distinctive synucleinopathies. To illustrate, the familial PD pathway that is caused by mutated α-synuclein may not be indistinguishable from those disturbed by alternative genes that are associated with PD and may also differ from those concerning Lewy body dementia and MSA [8]. The disparities could then be due to discrepancies in the characteristics of α-synuclein strains, diversified interactions of the protein, and even explicit types of affected cells. 

Subsequently, a plethora of pathways may be afflicted at differing points on the disease progression timelines of synucleinopathies [178]. Furthermore, certain pathways could be disturbed earlier in the pre-symptomatic stage in contrast to the later post-symptomatic states, while alternative pathways may atone for the defects in other pathways [8]. As such, varying pathways could have varied dysfunctional rates that might originally be the subthreshold for detection, until the degeneration of the cell has already occurred [8]. Nonetheless, subsequent factors, such as genetics and aging, may also alter the timing and specificity of pathway dysfunction [3,133,179]. 

The discrepancies in the alternative defective pathways examined in various studies could also be related to distinctions in α-synuclein’s experimental toxicity model [8]. To illustrate, the toxicity of α-synuclein is frequently modeled by upregulated WT α-synuclein or incubated or injected with preformed α-synuclein fibrils or oligomers, targeting α-synuclein expression utilizing adeno-associated virus vectors or PD-associated mutated α-synuclein [180,181]. The research studies are subsequently intricated due to the varying cell types chosen, such as neuronal, non-neuronal, or glial; the timeline of analysis; and even the animal model [180,181]. Certainly, utilizing differing models to elucidate the toxicity of α-synuclein, the varying steps in the production of autophagosomes, the fusion of the autophagosomes with lysosomes, and the degradative abilities of the mitochondria all possibly contribute to discrepancies [133,152,157,158]. Consequently, slight variabilities in the experimental preparation of oligomeric or fibrillated α-synuclein could generate strain disparities with differing propagation, seeding, and toxicity, further augmenting the possible variations noted in the observed cellular defects [129]. In further elucidation, α-synuclein mice models of toxicity allow for the analysis of operative neuromelanin, which is a crepuscular pigment encompassed by oxidized catecholamines like dopamine, which is a pivotal component of human dopaminergic substantia nigra pars compacta neurons [182,183]. Alternatively, dopaminergic neurons that were differentiated from human-derived induced pluripotent stem cells permit for a longer duration of human patient obtained cells with endogenous α-synuclein but are deficient in the intricate connections of the basal ganglia circuitry [149]. As such, further considerations and understanding of the capacities of the differing models and features are critical for the experimental design of the toxicity of α-synuclein, because they could reveal which synucleinopathies and what stages are the most appropriate for accurate reflections [8].

**Figure 3:**
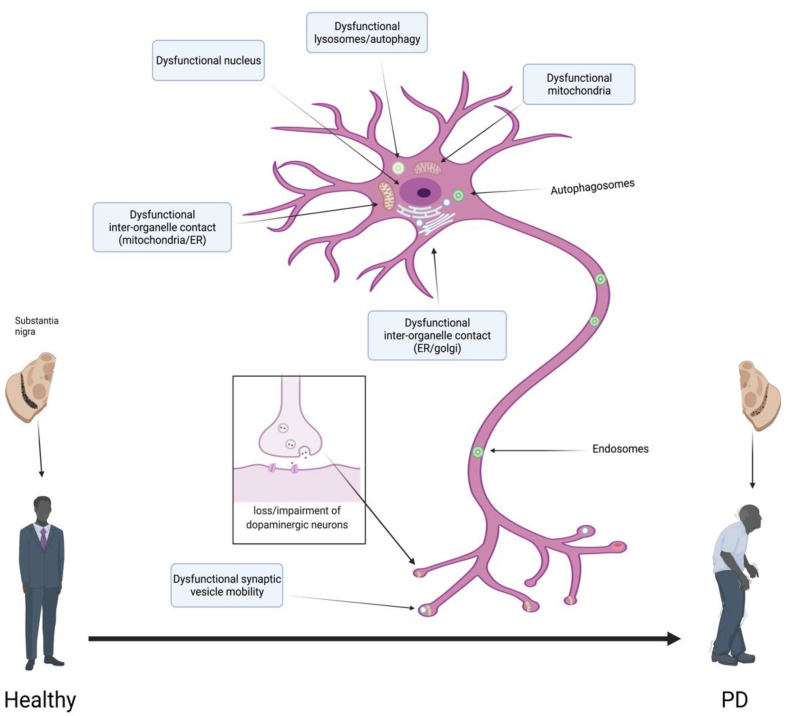
Pathways implicated in α-synuclein toxicity.

When examining the advancement of α-synuclein toxicity, although a plethora of pathways have been suspected in the downstream toxicity of α-synuclein (Figure 3), various supplementary variables may be critical for the extent, onset, and spread of the toxicity of α-synuclein [8]. To elucidate, post-mortem studies of humans have revealed that approximately 10–20% of the population displays incidental Lewy bodies without any clinically relevant neurological demonstration [184]. As such, contributing factors such as age could be critical for symptomatic determination, as well as severity and progression [62]. Nonetheless, it must be noted that the varying α-synuclein conformations, as well as strains, could subsequently affect α-synuclein toxicity [128]. Furthermore, alternate pathways have also been intricated in the boosting of the toxicity of α-synuclein in mouse and *Drosophila melanogaster* models, which incorporate the HSP70 deprivation, histone deacetylase sirtuin 2 incitement, and S129 phosphorylation of α-synuclein [50,185,186,187]. 

All things considered, aging seems to be the most critical variable for various neurodegenerative disorders, partly due to diminished functions of the organelles [188]. Neurons in the substantia nigra exhibit elevated deletion levels of mitochondrial DNA with age progression, leading to dysfunction of the mitochondria due to insufficiency of the respiratory chain [189]. Furthermore, the neuronal dysfunction of proteasomes also escalates with age progression due to the reduced expression of the proteasome subunits, as well as disassembly [190]. Even more, autophagy shows a reduced efficiency with age progression due to reduced levels of ATG5, ATG7, and beclin 1 autophagy proteins in the brains of humans [191]. As such, those defects could potentially aggravate the toxicity of α-synuclein, because dysfunctions in protein degradation could expedite the accumulation of α-synuclein. Evidently, the levels of α-synuclein are heightened in the substantia nigra of humans with the progression of age [188]. Furthermore, another factor that elevates with aging is oxidative stress, which leads to modifications of α-synuclein that are pathogenic, like nitration of the tyrosine residues, which have been seen in PD, MSA, and dementia with Lewy body-afflicted brains [58,192]. It is critical to note that the nitration of α-synuclein boosts its aggregation and reduces its lipid-binding capabilities [60]. Furthermore, autophagic vesicles in the dopaminergic neurons that contain neuromelanin and lipofuscin produced by oxidized catecholamines and iron-catalyzed oxidations also accrue with age progression [193]. 

Consequently, the actions of glia (microglia, oligodendrocytes, and astrocytes) have recently been studied more heavily in relation to neurodegeneration [8,194]. Nonetheless, even though α-synuclein accrues in the oligodendrocytes in MSA, where it is identified as the primary component of the glial cytoplasmic inclusions found in the disorder, it is uncharacterized if this occurs in differing synucleinopathies [130,195]. In general, glial cytoplasmic inclusions are critical microscopic hallmarks of MSA, along with α-synuclein aggregates that can also seemingly be detected in the neurons [196]. Likewise, involvement of the astrocytes in the toxicity of α-synuclein has also not been evidently decoded, even though astrocytes have the potential to obtain neuronally released α-synuclein through endocytosis, which causes changes in the genetic expression suggestive of inflammatory responses [194]. Consequently, the activation of microglial cells has also been witnessed in PD and MSA patients, implying that neuroinflammation could contribute to α-synuclein toxicity and pathogenesis [197]. Evidently, neuroinflammation has been detected in a plethora of α-synuclein animal models of toxicity and could be arbitrated by the microglial cell expression of MHC II, a prominent regulator of immune responses [198]. In general, MHC II depletion diminishes the activation of microglial cells, as well as the neurodegeneration of dopaminergic neurons in the mice models of α-synuclein toxicity [199]. Furthermore, the elevated expression of α-synuclein also has the potential to augment TLR4 immunoreactivity in mice models of MSA [197]. 

Numerous mechanisms have been suggested for the actuated neuroinflammatory response brought about by α-synuclein [8]. For example, extracellular endogenous α-synuclein discharged from neurons has been determined to serve as an agonist for TLR2, thereby activating microglial cells, while α-synuclein oligomers have been suggested to precisely bind TLR1/2 heterodimers to the cell membrane to potentiate an inflammatory response reliant on MyD88, which is a myeloid differentiation gene [200,201]. α-synuclein has likewise been proposed to serve as a chemoattractant that encourages the migration of microglial cells, and the inflammatory response derived from toxic α-synuclein could be arbitrated by miR-155 [202,203]. Consequently, dopaminergic neurons in PD patients could also be especially vulnerable to immune modulations, since mice that lack the IFN- cytokine generate a spontaneous degeneration of dopaminergic neurons [204]. Similarly, this also leads to Lewy body pathology, as well as cognitive and motor impairment. When observing MHC II, genetic polymorphisms have also been observed in HLA-DR, and this has been associated with a late onset of sporadic PD [205]. Thus, subsequent research on microglial cell activation and neuroinflammation in the toxicity of α-synuclein are then critical to establishing a clearer understanding of the pathophysiology of the protein that could further lead to the production of forthcoming synucleinopathy therapeutics. 

## 6. Examining the Interplay of α-Synuclein with Tau and Aβ

When observing AD patients, the reciprocity of amyloid beta (Aβ), tau, and α-synuclein can be considered. To illustrate, approximately fifty percent of AD patients seem to have Lewy body pathologies, and the levels of soluble α-synuclein are elevated in the brains of AD patients, which is associated with deteriorating cognitive function [206]. This could indicate that α-synuclein could potentially augment cholinergic and hippocampal neurodegeneration in the brains of AD patients [206]. Initial studies utilizing double-transgenic mice experimental models determined that Aβ supplemented the fibrilization of α-synuclein in vivo and in vitro, as well as that α-synuclein seemed to boost 1-38 Aβ aggregation when co-incubated in vitro [207,208]. Alternatively, injected fibrillated α-synuclein into transgenic mice models of AD were unsuccessful at cross-seeding Aβ in vivo, and the mice that co-expressed the A30P mutations of α-synuclein appeared to inhibit the formation of plaques in the mutant amyloid precursor protein (APP), as well as presenilin 1 [209]. These experimental discoveries propose that, instead of α-synuclein cross-seeding with tau, it actually appears to inhibit the deposition of Aβ, thereby lowering the formation of plaques in vivo [207]. Furthermore, when observing tauopathy in PD, the hyperphosphorylation of tau has also been determined to disintegrate from microtubules, thereby leading to neuronal dysfunction [210]. Hyperphosphorylated tau are prone to assemble into oligomers, eventually developing into filamentous neurofibrillary tangles [211,212]. In general, this is crucial, because hyperphosphorylated tau has been observed to interact with α-synuclein to boost fibrilization and aggregation, subsequently leading to axonal transport dysfunction and Lewy body formation [213]. Subsequent research will then be critical for observing if preventing the aggregation of Aβ will elevate the levels of the toxic oligomeric Aβ and lead to the dysfunction of neurons, in spite of a decline in the formation of plaques. 

## 7. Future Directions

It has been over two decades since the identification of α-synuclein as a genetically associated causative factor of PD and primary constituent of Lewy bodies [6,178]. This has led to various research studies on numerous dysfunctional pathways and post-translational modifications and their effects on propagation, oligomerization, and activation of the glia [11,179,197]. In turn, these studies have elucidated differing routes that could be utilized to therapeutically treat these neurodegenerative diseases and target α-synuclein [148]. 

Nonetheless, there is a plethora of objectives that must still be determined. To illustrate, a more definite timeline of disease and pathway dysfunction during synucleinopathy progression, the identification of native and toxic α-synuclein isoforms in healthy and affected subjects, and the characterization of α-synuclein’s propagation could be crucial. Consequently, since α-synuclein appears to be intertwined with Aβ and tau fibrillization, which are critical proteins in the pathogenesis of AD, further apprehension of the interactive roles of these three proteins is necessary [8]. Finally, continuing to analyze the effects that the post-translational modifications of α-synuclein have in PD progression will also aid in furthering the understanding of their pathology and disease progression, as well as potentially discovering novel therapeutic targets. 

## Figures and Tables

**Table 1:** Post-translational modifications of α-synuclein.

Post-Translational Modification	Amino Acid Residues
Phosphorylation	S129 [43]
Nitration	Y39, Y125, Y133, Y136 [60]
Ubiquitination	K10, K12, K21, K23, K34, K43, K96 [97]
Sumoylation	K96, K102 [98]
O-GlcNAcylation	T72, T75, T81, S87 [94]
Truncation	K58, K80, K97, V74 [104]
Dopamine	E83, Y_125_EMPS_129_ [105]

## Data Availability

Not applicable.

## Abbreviations

Parkinson’s disease: PD, amyloid precursor protein: APP, phospholipase D2: PLD2, phospholipase D: PLD, vesicle-associated membrane protein 2: VAMP2, reactive oxygen species: ROS, sodium dodecyl sulfate: SDS, seven in absentia homolog: SIAH, tripartite motif-containing 28: TRIM28, transcription factor EB (TFEB), and substantia pars nigra compacta: SNC.

## References

[1] Gitler A.D., Dhillon P., Shorter J. (2017). Neurodegenerative disease: Models, mechanisms, and a new hope. Dis. Model. Mech..

[2] Dorsey E.R., Elbaz A., Nichols E., Abd-Allah F., Abdelalim A., Adsuar J.C., Ansha G.M., Brayne C., Choi J.Y., Collado-Mateo D. (2018). Global, regional, and national burden of Parkinson’s disease, 1990–2016: A systematic analysis for the Global Burden of Disease Study 2016. Lancet Neurol..

[3] Balestrino R., Schapira A.H.V. (2020). Parkinson disease. Eur. J. Neurol..

[4] Váradi C. (2020). Clinical Features of Parkinson’s Disease: The Evolution of Critical Symptoms. Biology.

[5] Conway K.A., Rochet J.C., Bieganski R.M., Lansbury P.T. (2001). Kinetic stabilization of the alpha-synuclein protofibril by a dopamine-alpha-synuclein adduct. Science.

[6] Spillantini M.G., Crowther R.A., Jakes R., Hasegawa M., Goedert M. (1998). Alpha-Synuclein in filamentous inclusions of Lewy bodies from Parkinson’s disease and dementia with lewy bodies. Proc. Natl. Acad. Sci. USA.

[7] Polymeropoulos M.H., Lavedan C., Leroy E., Ide S.E., Dehejia A., Dutra A., Pike B., Root H., Rubenstein J., Boyer R. (1997). Mutation in the alpha-synuclein gene identified in families with Parkinson’s disease. Science.

[8] Wong Y.C., Krainc D. (2017). α-synuclein toxicity in neurodegeneration: Mechanism and therapeutic strategies. Nat. Med..

[9] Singleton A.B., Farrer M., Johnson J., Singleton A., Hague S., Kachergus J., Hulihan M., Peuralinna T., Dutra A., Nussbaum R. (2003). alpha-Synuclein locus triplication causes Parkinson’s disease. Science.

[10] Chartier-Harlin M.C., Kachergus J., Roumier C., Mouroux V., Douay X., Lincoln S., Levecque C., Larvor L., Andrieux J., Hulihan M. (2004). Alpha-synuclein locus duplication as a cause of familial Parkinson’s disease. Lancet.

[11] Zhang J., Li X., Li J.D. (2019). The Roles of Post-translational Modifications on α-Synuclein in the Pathogenesis of Parkinson’s Diseases. Front Neurosci..

[12] Maroteaux L., Campanelli J.T., Scheller R.H. (1988). Synuclein: A neuron-specific protein localized to the nucleus and presynaptic nerve terminal. J. Neurosci..

[13] Iwai A., Masliah E., Yoshimoto M., Ge N., Flanagan L., de Silva H.A., Kittel A., Saitoh T. (1995). The precursor protein of non-A beta component of Alzheimer’s disease amyloid is a presynaptic protein of the central nervous system. Neuron.

[14] Maroteaux L., Scheller R.H. (1991). The rat brain synucleins; family of proteins transiently associated with neuronal membrane. Brain Res. Mol. Brain Res..

[15] Bendor J.T., Logan T.P., Edwards R.H. (2013). The function of α-synuclein. Neuron.

[16] Dikiy I., Eliezer D. (2012). Folding and misfolding of alpha-synuclein on membranes. Biochim. Biophys. Acta.

[17] Prusiner S.B., Woerman A.L., Mordes D.A., Watts J.C., Rampersaud R., Berry D.B., Patel S., Oehler A., Lowe J.K., Kravitz S.N. (2015). Evidence for α-synuclein prions causing multiple system atrophy in humans with parkinsonism. Proc. Natl. Acad. Sci. USA.

[18] Woerman A.L., Stöhr J., Aoyagi A., Rampersaud R., Krejciova Z., Watts J.C., Ohyama T., Patel S., Widjaja K., Oehler A. (2015). Propagation of prions causing synucleinopathies in cultured cells. Proc. Natl. Acad. Sci. USA.

[19] Scott D., Roy S. (2012). α-Synuclein inhibits intersynaptic vesicle mobility and maintains recycling-pool homeostasis. J. Neurosci..

[20] Burré J., Sharma M., Südhof T.C. (2014). α-Synuclein assembles into higher-order multimers upon membrane binding to promote SNARE complex formation. Proc. Natl. Acad. Sci. USA.

[21] Nemani V.M., Lu W., Berge V., Nakamura K., Onoa B., Lee M.K., Chaudhry F.A., Nicoll R.A., Edwards R.H. (2010). Increased expression of alpha-synuclein reduces neurotransmitter release by inhibiting synaptic vesicle reclustering after endocytosis. Neuron.

[22] Alerte T.N., Akinfolarin A.A., Friedrich E.E., Mader S.A., Hong C.S., Perez R.G. (2008). Alpha-synuclein aggregation alters tyrosine hydroxylase phosphorylation and immunoreactivity: Lessons from viral transduction of knockout mice. Neurosci. Lett..

[23] Gorbatyuk O.S., Li S., Nguyen F.N., Manfredsson F.P., Kondrikova G., Sullivan L.F., Meyers C., Chen W., Mandel R.J., Muzyczka N. (2010). α-Synuclein expression in rat substantia nigra suppresses phospholipase D2 toxicity and nigral neurodegeneration. Mol. Ther..

[24] McDermott M., Wakelam M.J., Morris A.J. (2004). Phospholipase D. Biochem. Cell Biol..

[25] Rappley I., Gitler A.D., Selvy P.E., LaVoie M.J., Levy B.D., Brown H.A., Lindquist S., Selkoe D.J. (2009). Evidence that alpha-synuclein does not inhibit phospholipase D. Biochemistry.

[26] Emmanouilidou E., Melachroinou K., Roumeliotis T., Garbis S.D., Ntzouni M., Margaritis L.H., Stefanis L., Vekrellis K. (2010). Cell-produced alpha-synuclein is secreted in a calcium-dependent manner by exosomes and impacts neuronal survival. J. Neurosci..

[27] El-Agnaf O.M., Salem S.A., Paleologou K.E., Cooper L.J., Fullwood N.J., Gibson M.J., Curran M.D., Court J.A., Mann D.M., Ikeda S. (2003). Alpha-synuclein implicated in Parkinson’s disease is present in extracellular biological fluids, including human plasma. FASEB J..

[28] El-Agnaf O.M., Salem S.A., Paleologou K.E., Curran M.D., Gibson M.J., Court J.A., Schlossmacher M.G., Allsop D. (2006). Detection of oligomeric forms of alpha-synuclein protein in human plasma as a potential biomarker for Parkinson’s disease. FASEB J..

[29] Emmanouilidou E., Elenis D., Papasilekas T., Stranjalis G., Gerozissis K., Ioannou P.C., Vekrellis K. (2011). Assessment of α-synuclein secretion in mouse and human brain parenchyma. PLoS ONE.

[30] Lee H.J., Patel S., Lee S.J. (2005). Intravesicular localization and exocytosis of alpha-synuclein and its aggregates. J. Neurosci..

[31] Sung J.Y., Park S.M., Lee C.H., Um J.W., Lee H.J., Kim J., Oh Y.J., Lee S.T., Paik S.R., Chung K.C. (2005). Proteolytic cleavage of extracellular secreted {alpha}-synuclein via matrix metalloproteinases. J. Biol. Chem..

[32] Van Niel G., Porto-Carreiro I., Simoes S., Raposo G. (2006). Exosomes: A common pathway for a specialized function. J. Biochem..

[33] Vincent J.P., Magee T. (2002). Argosomes: Membrane fragments on the run. Trends Cell Biol..

[34] Théry C., Zitvogel L., Amigorena S. (2002). Exosomes: Composition, biogenesis and function. Nat. Rev. Immunol..

[35] Mehul B., Hughes R.C. (1997). Plasma membrane targetting, vesicular budding and release of galectin 3 from the cytoplasm of mammalian cells during secretion. J. Cell Sci..

[36] Hughes R.C. (1999). Secretion of the galectin family of mammalian carbohydrate–binding proteins. Biochim. Biophys. Acta.

[37] Krüger R., Kuhn W., Müller T., Woitalla D., Graeber M., Kösel S., Przuntek H., Epplen J.T., Schöls L., Riess O. (1998). Ala30Pro mutation in the gene encoding alpha-synuclein in Parkinson’s disease. Nat. Genet..

[38] Iacono D., Geraci-Erck M., Rabin M.L., Adler C.H., Serrano G., Beach T.G., Kurlan R. (2015). Parkinson disease and incidental Lewy body disease: Just a question of time?. Neurology.

[39] Fagerqvist T., Lindström V., Nordström E., Lord A., Tucker S.M., Su X., Sahlin C., Kasrayan A., Andersson J., Welander H. (2013). Monoclonal antibodies selective for α-synuclein oligomers/protofibrils recognize brain pathology in Lewy body disorders and α-synuclein transgenic mice with the disease-causing A30P mutation. J. Neurochem..

[40] Gómez-Benito M., Granado N., García-Sanz P., Michel A., Dumoulin M., Moratalla R. (2020). Modeling Parkinson’s Disease With the Alpha-Synuclein Protein. Front. Pharmacol..

[41] Fujiwara H., Hasegawa M., Dohmae N., Kawashima A., Masliah E., Goldberg M.S., Shen J., Takio K., Iwatsubo T. (2002). Alpha-Synuclein is phosphorylated in synucleinopathy lesions. Nat. Cell. Biol..

[42] Muntané G., Ferrer I., Martinez-Vicente M. (2012). α-synuclein phosphorylation and truncation are normal events in the adult human brain. Neuroscience.

[43] Oueslati A. (2016). Implication of Alpha-Synuclein Phosphorylation at S129 in Synucleinopathies: What Have We Learned in the Last Decade?. J. Parkinsons Dis..

[44] Barrett P.J., Timothy G.J. (2015). Post-translational modification of α-synuclein in Parkinson’s disease. Brain Res..

[45] Schell H., Hasegawa T., Neumann M., Kahle P.J. (2009). Nuclear and neuritic distribution of serine-129 phosphorylated alpha-synuclein in transgenic mice. Neuroscience.

[46] Sato H., Kato T., Arawaka S. (2013). The role of Ser129 phosphorylation of α-synuclein in neurodegeneration of Parkinson’s disease: A review of in vivo models. Rev. Neurosci..

[47] Smith W.W., Margolis R.L., Li X., Troncoso J.C., Lee M.K., Dawson V.L., Dawson T.M., Iwatsubo T., Ross C.A. (2005). Alpha-synuclein phosphorylation enhances eosinophilic cytoplasmic inclusion formation in SH-SY5Y cells. J. Neurosci..

[48] Mbefo M.K., Paleologou K.E., Boucharaba A., Oueslati A., Schell H., Fournier M., Olschewski D., Yin G., Zweckstetter M., Masliah E. (2010). Phosphorylation of synucleins by members of the Polo-like kinase family. J. Biol. Chem..

[49] Azeredo da Silveira S., Schneider B.L., Cifuentes-Diaz C., Sage D., Abbas-Terki T., Iwatsubo T., Unser M., Aebischer P. (2009). Phosphorylation does not prompt, nor prevent, the formation of alpha-synuclein toxic species in a rat model of Parkinson’s disease. Hum. Mol. Genet..

[50] Chen L., Feany M.B. (2005). Alpha-synuclein phosphorylation controls neurotoxicity and inclusion formation in a Drosophila model of Parkinson disease. Nat. Neurosci..

[51] Chen L., Periquet M., Wang X., Negro A., McLean P.J., Hyman B.T., Feany M.B. (2009). Tyrosine and serine phosphorylation of alpha-synuclein have opposing effects on neurotoxicity and soluble oligomer formation. J. Clin. Investig..

[52] Luth E.S., Stavrovskaya I.G., Bartels T., Kristal B.S., Selkoe D.J. (2014). Soluble, prefibrillar α-synuclein oligomers promote complex I-dependent, Ca2+-induced mitochondrial dysfunction. J. Biol. Chem..

[53] Robin M.A., Prabu S.K., Raza H., Anandatheerthavarada H.K., Avadhani N.G. (2003). Phosphorylation enhances mitochondrial targeting of GSTA4-4 through increased affinity for binding to cytoplasmic Hsp70. J. Biol. Chem..

[54] Linseman D.A., Butts B.D., Precht T.A., Phelps R.A., Le S.S., Laessig T.A., Bouchard R.J., Florez-McClure M.L., Heidenreich K.A. (2004). Glycogen synthase kinase-3beta phosphorylates Bax and promotes its mitochondrial localization during neuronal apoptosis. J. Neurosci..

[55] Moreira P.I., Sayre L.M., Zhu X., Nunomura A., Smith M.A., Perry G. (2010). Detection and localization of markers of oxidative stress by in situ methods: Application in the study of Alzheimer disease. Methods Mol. Biol..

[56] Perfeito R., Lázaro D.F., Outeiro T.F., Rego A.C. (2014). Linking alpha-synuclein phosphorylation to reactive oxygen species formation and mitochondrial dysfunction in SH-SY5Y cells. Mol. Cell. Neurosci..

[57] Good P.F., Hsu A., Werner P., Perl D.P., Olanow C.W. (1998). Protein nitration in Parkinson’s disease. J. Neuropathol Exp. Neurol..

[58] Giasson B.I., Duda J.E., Murray I.V., Chen Q., Souza J.M., Hurtig H.I., Ischiropoulos H., Trojanowski J.Q., Lee V.M. (2000). Oxidative damage linked to neurodegeneration by selective alpha-synuclein nitration in synucleinopathy lesions. Science.

[59] Al-Hilaly Y.K., Biasetti L., Blakeman B.J., Pollack S.J., Zibaee S., Abdul-Sada A., Thorpe J.R., Xue W.F., Serpell L.C. (2016). The involvement of dityrosine crosslinking in α-synuclein assembly and deposition in Lewy Bodies in Parkinson’s disease. Sci. Rep..

[60] Hodara R., Norris E.H., Giasson B.I., Mishizen-Eberz A.J., Lynch D.R., Lee V.M., Ischiropoulos H. (2004). Functional consequences of alpha-synuclein tyrosine nitration: Diminished binding to lipid vesicles and increased fibril formation. J. Biol. Chem..

[61] Bell R., Vendruscolo M. (2021). Modulation of the Interactions Between α-Synuclein and Lipid Membranes by Post-translational Modifications. Front Neurol..

[62] Stefanis L. (2012). α-Synuclein in Parkinson’s disease. Cold Spring Harb. Perspect. Med..

[63] Dias V., Junn E., Mouradian M.M. (2013). The role of oxidative stress in Parkinson’s disease. J. Parkinsons Dis..

[64] Hwang O. (2013). Role of oxidative stress in Parkinson’s disease. Exp. Neurobiol..

[65] Cui H., Kong Y., Zhang H. (2012). Oxidative stress, mitochondrial dysfunction, and aging. J. Signal Transduct..

[66] Huang M.L., Chiang S., Kalinowski D.S., Bae D.H., Sahni S., Richardson D.R. (2019). The Role of the Antioxidant Response in Mitochondrial Dysfunction in Degenerative Diseases: Cross-Talk between Antioxidant Defense, Autophagy, and Apoptosis. Oxid Med. Cell Longev..

[67] Oliveira L.M.A., Gasser T., Edwards R., Zweckstetter M., Melki R., Stefanis L., Lashuel H.A., Sulzer D., Vekrellis K., Halliday G.M. (2021). Alpha-synuclein research: Defining strategic moves in the battle against Parkinson’s disease. NPJ Parkinsons Dis..

[68] Juárez Olguín H., Calderón Guzmán D., Hernández García E., Barragán Mejía G. (2016). The Role of Dopamine and Its Dysfunction as a Consequence of Oxidative Stress. Oxid. Med. Cell. Longev..

[69] Asanuma M., Miyazaki I., Ogawa N. (2003). Dopamine- or L-DOPA-induced neurotoxicity: The role of dopamine quinone formation and tyrosinase in a model of Parkinson’s disease. Neurotox. Res..

[70] Ballatori N., Krance S.M., Notenboom S., Shi S., Tieu K., Hammond C.L. (2009). Glutathione dysregulation and the etiology and progression of human diseases. Biol. Chem..

[71] Martin H.L., Teismann P. (2009). Glutathione--a review on its role and significance in Parkinson’s disease. FASEB J..

[72] Lotharius J., Barg S., Wiekop P., Lundberg C., Raymon H.K., Brundin P. (2002). Effect of mutant alpha-synuclein on dopamine homeostasis in a new human mesencephalic cell line. J. Biol. Chem..

[73] Umek N., Geršak B., Vintar N., Šoštarič M., Mavri J. (2018). Dopamine Autoxidation Is Controlled by Acidic pH. Front. Mol. Neurosci..

[74] Ingelsson M. (2016). Alpha-Synuclein Oligomers-Neurotoxic Molecules in Parkinson’s Disease and Other Lewy Body Disorders. Front Neurosci..

[75] Emanuele M., Chieregatti E. (2015). Mechanisms of alpha-synuclein action on neurotransmission: Cell-autonomous and non-cell autonomous role. Biomolecules.

[76] German C.L., Baladi M.G., McFadden L.M., Hanson G.R., Fleckenstein A.E. (2015). Regulation of the Dopamine and Vesicular Monoamine Transporters: Pharmacological Targets and Implications for Disease. Pharmacol. Rev..

[77] Tabrez S., Jabir N.R., Shakil S., Greig N.H., Alam Q., Abuzenadah A.M., Damanhouri G.A., Kamal M.A. (2012). A synopsis on the role of tyrosine hydroxylase in Parkinson’s disease. CNS Neurol. Disord. Drug Targets.

[78] Rantham Prabhakara J.P., Feist G., Thomasson S., Thompson A., Schommer E., Ghribi O. (2008). Differential effects of 24-hydroxycholesterol and 27-hydroxycholesterol on tyrosine hydroxylase and alpha-synuclein in human neuroblastoma SH-SY5Y cells. J. Neurochem..

[79] Perez R.G., Waymire J.C., Lin E., Liu J.J., Guo F., Zigmond M.J. (2002). A role for alpha-synuclein in the regulation of dopamine biosynthesis. J. Neurosci..

[80] Mor D.E., Ugras S.E., Daniels M.J., Ischiropoulos H. (2016). Dynamic structural flexibility of α-synuclein. Neurobiol. Dis..

[81] Dehay B., Bourdenx M., Gorry P., Przedborski S., Vila M., Hunot S., Singleton A., Olanow C.W., Merchant K.M., Bezard E. (2015). Targeting α-synuclein for treatment of Parkinson’s disease: Mechanistic and therapeutic considerations. Lancet Neurol..

[82] Li J., Zhu M., Manning-Bog A.B., Di Monte D.A., Fink A.L. (2004). Dopamine and L-dopa disaggregate amyloid fibrils: Implications for Parkinson’s and Alzheimer’s disease. FASEB J..

[83] Cappa i.R., Leck S.L., Tew D.J., Williamson N.A., Smith D.P., Galatis D., Sharples R.A., Curtain C.C., Ali F.E., Cherny R.A. (2005). Dopamine promotes alpha-synuclein aggregation into SDS-resistant soluble oligomers via a distinct folding pathway. FASEB J..

[84] Ariesandi W., Chang C.F., Chen T.E., Chen Y.R. (2013). Temperature-dependent structural changes of Parkinson’s alpha-synuclein reveal the role of pre-existing oligomers in alpha-synuclein fibrillization. PLoS ONE.

[85] Pham C.L., Cappai R. (2013). The interplay between lipids and dopamine on α-synuclein oligomerization and membrane binding. Biosci. Rep..

[86] Luth E.S., Bartels T., Dettmer U., Kim N.C., Selkoe D.J. (2015). Purification of α-synuclein from human brain reveals an instability of endogenous multimers as the protein approaches purity. Biochemistry.

[87] Mor D.E., Daniels M.J., Ischiropoulos H. (2019). The usual suspects, dopamine and alpha-synuclein, conspire to cause neurodegeneration. Mov. Disord..

[88] Maguire-Zeiss K.A., Short D.W., Federoff H.J. (2005). Synuclein, dopamine and oxidative stress: Co-conspirators in Parkinson’s disease?. Brain Res. Mol. Brain Res..

[89] Sulzer D. (2010). Clues to how alpha-synuclein damages neurons in Parkinson’s disease. Mov. Disord..

[90] He S., Wang F., Yung K.K.L., Zhang S., Qu S. (2021). Effects of α-Synuclein-Associated Post-Translational Modifications in Parkinson’s Disease. ACS Chem. Neurosci..

[91] Volles M.J., Lansbury P.T. (2002). Vesicle permeabilization by protofibrillar alpha-synuclein is sensitive to Parkinson’s disease-linked mutations and occurs by a pore-like mechanism. Biochemistry.

[92] Luk K.C., Kehm V., Carroll J., Zhang B., O’Brien P., Trojanowski J.Q., Lee V.M. (2012). Pathological α-synuclein transmission initiates Parkinson-like neurodegeneration in nontransgenic mice. Science.

[93] Hashimoto M., Rockenstein E., Crews L., Masliah E. (2003). Role of protein aggregation in mitochondrial dysfunction and neurodegeneration in Alzheimer’s and Parkinson’s diseases. Neuromolecular. Med..

[94] Levine P.M., Galesic A., Balana A.T., Mahul-Mellier A.L., Navarro M.X., De Leon C.A., Lashuel H.A., Pratt M.R. (2019). α-Synuclein O-GlcNAcylation alters aggregation and toxicity, revealing certain residues as potential inhibitors of Parkinson’s disease. Proc. Natl. Acad. Sci. USA.

[95] Krumova P., Meulmeester E., Garrido M., Tirard M., Hsiao H.H., Bossis G., Urlaub H., Zweckstetter M., Kügler S., Melchior F. (2011). Sumoylation inhibits alpha-synuclein aggregation and toxicity. J. Cell Biol..

[96] Savyon M., Engelender S. (2020). SUMOylation in α-Synuclein Homeostasis and Pathology. Front. Aging Neurosci..

[97] Lee J.T., Wheeler T.C., Li L., Chin L.S. (2008). Ubiquitination of alpha-synuclein by Siah-1 promotes alpha-synuclein aggregation and apoptotic cell death. Hum. Mol. Genet..

[98] Rott R., Szargel R., Shani V., Hamza H., Savyon M., Abd Elghani F., Bandopadhyay R., Engelender S. (2017). SUMOylation and ubiquitination reciprocally regulate α-synuclein degradation and pathological aggregation. Proc. Natl Acad. Sci. USA.

[99] Baba M., Nakajo S., Tu P.H., Tomita T., Nakaya K., Lee V.M., Trojanowski J.Q., Iwatsubo T. (1998). Aggregation of alpha-synuclein in Lewy bodies of sporadic Parkinson’s disease and dementia with Lewy bodies. Am. J. Pathol..

[100] Campbell B.C., McLean C.A., Culvenor J.G., Gai W.P., Blumbergs P.C., Jäkälä P., Beyreuther K., Masters C.L., Li Q.X. (2001). The solubility of alpha-synuclein in multiple system atrophy differs from that of dementia with Lewy bodies and Parkinson’s disease. J. Neurochem..

[101] Kasai T., Tokuda T., Yamaguchi N., Watanabe Y., Kametani F., Nakagawa M., Mizuno T. (2008). Cleavage of normal and pathological forms of alpha-synuclein by neurosin in vitro. Neurosci. Lett..

[102] Mishizen-Eberz A.J., Norris E.H., Giasson B.I., Hodara R., Ischiropoulos H., Lee V.M., Trojanowski J.Q., Lynch D.R. (2005). Cleavage of alpha-synuclein by calpain: Potential role in degradation of fibrillized and nitrated species of alpha-synuclein. Biochemistry.

[103] Iwata A., Maruyama M., Akagi T., Hashikawa T., Kanazawa I., Tsuji S., Nukina N. (2003). Alpha-synuclein degradation by serine protease neurosin: Implication for pathogenesis of synucleinopathies. Hum. Mol. Genet..

[104] Mishizen-Eberz A.J., Guttmann R.P., Giasson B.I., Day G.A., Hodara R., Ischiropoulos H., Lee V.M., Trojanowski J.Q., Lynch D.R. (2003). Distinct cleavage patterns of normal and pathologic forms of alpha-synuclein by calpain I in vitro. J. Neurochem..

[105] Mor D.E., Tsika E., Mazzulli J.R., Gould N.S., Kim H., Daniels M.J., Doshi S., Gupta P., Grossman J.L., Tan V.X. (2017). Dopamine induces soluble α-synuclein oligomers and nigrostriatal degeneration. Nat. Neurosci..

[106] Deleersnijder A., Gerard M., Debyser Z., Baekelandt V. (2013). The remarkable conformational plasticity of alpha-synuclein: Blessing or curse?. Trends Mol. Med..

[107] Weinreb P.H., Zhen W., Poon A.W., Conway K.A., Lansbury P.T. (1996). NACP, a protein implicated in Alzheimer’s disease and learning, is natively unfolded. Biochemistry.

[108] Dettmer U., Newman A.J., von Saucken V.E., Bartels T., Selkoe D. (2015). KTKEGV repeat motifs are key mediators of normal α-synuclein tetramerization: Their mutation causes excess monomers and neurotoxicity. Proc. Natl. Acad. Sci. USA.

[109] Lemkau L.R., Comellas G., Lee S.W., Rikardsen L.K., Woods W.S., George J.M., Rienstra C.M. (2013). Site-specific perturbations of alpha-synuclein fibril structure by the Parkinson’s disease associated mutations A53T and E46K. PLoS ONE.

[110] Zarranz J.J., Alegre J., Gómez-Esteban J.C., Lezcano E., Ros R., Ampuero I., Vidal L., Hoenicka J., Rodriguez O., Atarés B. (2004). The new mutation, E46K, of alpha-synuclein causes Parkinson and Lewy body dementia. Ann. Neurol..

[111] Cascella R., Chen S.W., Bigi A., Camino J.D., Xu C.K., Dobson C.M., Chiti F., Cremades N., Cecchi C. (2021). The release of toxic oligomers from α-synuclein fibrils induces dysfunction in neuronal cells. Nat. Commun..

[112] Assayag K., Yakunin E., Loeb V., Selkoe D.J., Sharon R. (2007). Polyunsaturated fatty acids induce alpha-synuclein-related pathogenic changes in neuronal cells. Am. J. Pathol..

[113] Sharon R., Bar-Joseph I., Frosch M.P., Walsh D.M., Hamilton J.A., Selkoe D.J. (2003). The formation of highly soluble oligomers of alpha-synuclein is regulated by fatty acids and enhanced in Parkinson’s disease. Neuron.

[114] Tanji K., Mori F., Mimura J., Itoh K., Kakita A., Takahashi H. (2010). Proteinase K-resistant alpha-synuclein is deposited in presynapses in human Lewy body disease and A53T alpha-synuclein transgenic mice. Acta Neuropathol..

[115] Snead D., Eliezer D. (2014). Alpha-synuclein function and dysfunction on cellular membranes. Exp. Neurobiol..

[116] Perrin R.J., Woods W.S., Clayton D.F., George J.M. (2001). Exposure to long chain polyunsaturated fatty acids triggers rapid multimerization of synucleins. J. Biol. Chem..

[117] Ferreon A.C., Gambin Y., Lemke E.A., Deniz A.A. (2009). Interplay of alpha-synuclein binding and conformational switching probed by single-molecule fluorescence. Proc. Natl. Acad. Sci. USA.

[118] Buell A.K., Galvagnion C., Gaspar R., Sparr E., Vendruscolo M., Knowles T.P., Linse S., Dobson C.M. (2014). Solution conditions determine the relative importance of nucleation and growth processes in α-synuclein aggregation. Proc. Natl. Acad. Sci. USA.

[119] Galvagnion C., Buell A.K., Meisl G., Michaels T.C., Vendruscolo M., Knowles T.P., Dobson C.M. (2015). Lipid vesicles trigger α-synuclein aggregation by stimulating primary nucleation. Nat. Chem. Biol..

[120] Lashuel H.A., Overk C.R., Oueslati A., Masliah E. (2013). The many faces of α-synuclein: From structure and toxicity to therapeutic target. Nat. Rev. Neurosci..

[121] Winner B., Jappelli R., Maji S.K., Desplats P.A., Boyer L., Aigner S., Hetzer C., Loher T., Vilar M., Campioni S. (2011). In vivo demonstration that alpha-synuclein oligomers are toxic. Proc. Natl. Acad. Sci. USA.

[122] Pasanen P., Myllykangas L., Siitonen M., Raunio A., Kaakkola S., Lyytinen J., Tienari P.J., Pöyhönen M., Paetau A. (2014). Novel α-synuclein mutation A53E associated with atypical multiple system atrophy and Parkinson’s disease-type pathology. Neurobiol. Aging.

[123] Pieri L., Madiona K., Bousset L., Melki R. (2012). Fibrillar α-synuclein and huntingtin exon 1 assemblies are toxic to the cells. Biophys. J..

[124] Ghiglieri V., Calabrese V., Calabresi P. (2018). Alpha-Synuclein: From Early Synaptic Dysfunction to Neurodegeneration. Front. Neurol..

[125] Alam P., Bousset L., Melki R., Otzen D.E. (2019). α-synuclein oligomers and fibrils: A spectrum of species, a spectrum of toxicities. J. Neurochem..

[126] Kumar S.T., Jagannath S., Francois C., Vanderstichele H., Stoops E., Lashuel H.A. (2020). How specific are the conformation-specific α-synuclein antibodies? Characterization and validation of 16 α-synuclein conformation-specific antibodies using well-characterized preparations of α-synuclein monomers, fibrils and oligomers with distinct structures and morphology. Neurobiol. Dis..

[127] Fields C.R., Bengoa-Vergniory N., Wade-Martins R. (2019). Targeting Alpha-Synuclein as a Therapy for Parkinson’s Disease. Front. Mol. Neurosci..

[128] Bousset L., Pieri L., Ruiz-Arlandis G., Gath J., Jensen P.H., Habenstein B., Madiona K., Olieric V., Böckmann A., Meier B.H. (2013). Structural and functional characterization of two alpha-synuclein strains. Nat. Commun..

[129] Guo J.L., Covell D.J., Daniels J.P., Iba M., Stieber A., Zhang B., Riddle D.M., Kwong L.K., Xu Y., Trojanowski J.Q. (2013). Distinct α-synuclein strains differentially promote tau inclusions in neurons. Cell.

[130] Spillantini M.G., Crowther R.A., Jakes R., Cairns N.J., Lantos P.L., Goedert M. (1998). Filamentous alpha-synuclein inclusions link multiple system atrophy with Parkinson’s disease and dementia with Lewy bodies. Neurosci. Lett..

[131] Rodriguez J.A., Ivanova M.I., Sawaya M.R., Cascio D., Reyes F.E., Shi D., Sangwan S., Guenther E.L., Johnson L.M., Zhang M. (2015). Structure of the toxic core of α-synuclein from invisible crystals. Nature.

[132] Outeiro T.F., Lindquist S. (2003). Yeast cells provide insight into alpha-synuclein biology and pathobiology. Science.

[133] Volpicelli-Daley L.A., Gamble K.L., Schultheiss C.E., Riddle D.M., West A.B., Lee V.M. (2014). Formation of α-synuclein Lewy neurite-like aggregates in axons impedes the transport of distinct endosomes. Mol. Biol. Cell.

[134] Volpicelli-Daley L.A. (2017). Effects of α-synuclein on axonal transport. Neurobiol. Dis..

[135] Varkey J., Isas J.M., Mizuno N., Jensen M.B., Bhatia V.K., Jao C.C., Petrlova J., Voss J.C., Stamou D.G., Steven A.C. (2010). Membrane curvature induction and tubulation are common features of synucleins and apolipoproteins. J. Biol. Chem..

[136] Davidson W.S., Jonas A., Clayton D.F., George J.M. (1998). Stabilization of alpha-synuclein secondary structure upon binding to synthetic membranes. J. Biol. Chem..

[137] Chandra S., Gallardo G., Fernández-Chacón R., Schlüter O.M., Südhof T.C. (2005). Alpha-synuclein cooperates with CSPalpha in preventing neurodegeneration. Cell.

[138] Eisbach S.E., Outeiro T.F. (2013). Alpha-synuclein and intracellular trafficking: Impact on the spreading of Parkinson’s disease pathology. J. Mol. Med..

[139] Abeliovich A., Schmitz Y., Fariñas I., Choi-Lundberg D., Ho W.H., Castillo P.E., Shinsky N., Verdugo J.M., Armanini M., Ryan A. (2000). Mice lacking alpha-synuclein display functional deficits in the nigrostriatal dopamine system. Neuron.

[140] Lundblad M., Decressac M., Mattsson B., Björklund A. (2012). Impaired neurotransmission caused by overexpression of α-synuclein in nigral dopamine neurons. Proc. Natl. Acad. Sci. USA.

[141] Masliah E., Rockenstein E., Veinbergs I., Mallory M., Hashimoto M., Takeda A., Sagara Y., Sisk A., Mucke L. (2000). Dopaminergic loss and inclusion body formation in alpha-synuclein mice: Implications for neurodegenerative disorders. Science.

[142] Janezic S., Threlfell S., Dodson P.D., Dowie M.J., Taylor T.N., Potgieter D., Parkkinen L., Senior S.L., Anwar S., Ryan B. (2013). Deficits in dopaminergic transmission precede neuron loss and dysfunction in a new Parkinson model. Proc. Natl. Acad. Sci. USA.

[143] Lotharius J., Brundin P. (2002). Pathogenesis of Parkinson’s disease: Dopamine, vesicles and alpha-synuclein. Nat. Rev. Neurosci..

[144] Chen L., Xie Z., Turkson S., Zhuang X. (2015). A53T human α-synuclein overexpression in transgenic mice induces pervasive mitochondria macroautophagy defects preceding dopamine neuron degeneration. J. Neurosci..

[145] Nakamura K., Nemani V.M., Azarbal F., Skibinski G., Levy J.M., Egami K., Munishkina L., Zhang J., Gardner B., Wakabayashi J. (2011). Direct membrane association drives mitochondrial fission by the Parkinson disease-associated protein alpha-synuclein. J. Biol. Chem..

[146] Dauer W., Kholodilov N., Vila M., Trillat A.C., Goodchild R., Larsen K.E., Staal R., Tieu K., Schmitz Y., Yuan C.A. (2002). Resistance of alpha -synuclein null mice to the parkinsonian neurotoxin MPTP. Proc. Natl. Acad. Sci. USA.

[147] Di Maio R., Barrett P.J., Hoffman E.K., Barrett C.W., Zharikov A., Borah A., Hu X., McCoy J., Chu C.T., Burton E.A. (2016). α-Synuclein binds to TOM20 and inhibits mitochondrial protein import in Parkinson’s disease. Sci. Transl. Med..

[148] Zheng B., Liao Z., Locascio J.J., Lesniak K.A., Roderick S.S., Watt M.L., Eklund A.C., Zhang-James Y., Kim P.D., Hauser M.A. (2010). PGC-1α, a potential therapeutic target for early intervention in Parkinson’s disease. Sci. Transl. Med..

[149] Ryan S.D., Dolatabadi N., Chan S.F., Zhang X., Akhtar M.W., Parker J., Soldner F., Sunico C.R., Nagar S., Talantova M. (2013). Isogenic human iPSC Parkinson’s model shows nitrosative stress-induced dysfunction in MEF2-PGC1α transcription. Cell.

[150] Tardiff D.F., Jui N.T., Khurana V., Tambe M.A., Thompson M.L., Chung C.Y., Kamadurai H.B., Kim H.T., Lancaster A.K., Caldwell K.A. (2013). Yeast reveal a "druggable" Rsp5/Nedd4 network that ameliorates α-synuclein toxicity in neurons. Science.

[151] Cooper A.A., Gitler A.D., Cashikar A., Haynes C.M., Hill K.J., Bhullar B., Liu K., Xu K., Strathearn K.E., Liu F. (2006). Alpha-synuclein blocks ER-Golgi traffic and Rab1 rescues neuron loss in Parkinson’s models. Science.

[152] Chung C.Y., Khurana V., Auluck P.K., Tardiff D.F., Mazzulli J.R., Soldner F., Baru V., Lou Y., Freyzon Y., Cho S. (2013). Identification and rescue of α-synuclein toxicity in Parkinson patient-derived neurons. Science.

[153] Caraveo G., Auluck P.K., Whitesell L., Chung C.Y., Baru V., Mosharov E.V., Yan X., Ben-Johny M., Soste M., Picotti P. (2014). Calcineurin determines toxic versus beneficial responses to α-synuclein. Proc. Natl. Acad. Sci. USA.

[154] Bellucci A., Navarria L., Zaltieri M., Falarti E., Bodei S., Sigala S., Battistin L., Spillantini M., Missale C., Spano P. (2011). Induction of the unfolded protein response by α-synuclein in experimental models of Parkinson’s disease. J. Neurochem..

[155] Colla E., Jensen P.H., Pletnikova O., Troncoso J.C., Glabe C., Lee M.K. (2012). Accumulation of toxic α-synuclein oligomer within endoplasmic reticulum occurs in α-synucleinopathy in vivo. J. Neurosci..

[156] Mizushima N. (2007). Autophagy: Process and function. Genes Dev..

[157] Winslow A.R., Chen C.W., Corrochano S., Acevedo-Arozena A., Gordon D.E., Peden A.A., Lichtenberg M., Menzies F.M., Ravikumar B., Imarisio S. (2010). α-Synuclein impairs macroautophagy: Implications for Parkinson’s disease. J. Cell Biol..

[158] Cuervo A.M., Stefanis L., Fredenburg R., Lansbury P.T., Sulzer D. (2004). Impaired degradation of mutant alpha-synuclein by chaperone-mediated autophagy. Science.

[159] Martinez-Vicente M., Talloczy Z., Kaushik S., Massey A.C., Mazzulli J., Mosharov E.V., Hodara R., Fredenburg R., Wu D.C., Follenzi A. (2008). Dopamine-modified alpha-synuclein blocks chaperone-mediated autophagy. J. Clin. Investig..

[160] Mazzulli J.R., Xu Y.H., Sun Y., Knight A.L., McLean P.J., Caldwell G.A., Sidransky E., Grabowski G.A., Krainc D. (2011). Gaucher disease glucocerebrosidase and α-synuclein form a bidirectional pathogenic loop in synucleinopathies. Cell.

[161] Mazzulli J.R., Zunke F., Isacson O., Studer L., Krainc D. (2016). α-Synuclein-induced lysosomal dysfunction occurs through disruptions in protein trafficking in human midbrain synucleinopathy models. Proc. Natl. Acad. Sci. USA.

[162] Rousseaux M.W., de Haro M., Lasagna-Reeves C.A., De Maio A., Park J., Jafar-Nejad P., Al-Ramahi I., Sharma A., See L., Lu N. (2016). TRIM28 regulates the nuclear accumulation and toxicity of both alpha-synuclein and tau. eLife.

[163] Kontopoulos E., Parvin J.D., Feany M.B. (2006). Alpha-synuclein acts in the nucleus to inhibit histone acetylation and promote neurotoxicity. Hum. Mol. Genet..

[164] Fares M.B., Ait-Bouziad N., Dikiy I., Mbefo M.K., Jovičić A., Kiely A., Holton J.L., Lee S.J., Gitler A.D., Eliezer D. (2014). The novel Parkinson’s disease linked mutation G51D attenuates in vitro aggregation and membrane binding of α-synuclein, and enhances its secretion and nuclear localization in cells. Hum. Mol. Genet..

[165] Decressac M., Mattsson B., Weikop P., Lundblad M., Jakobsson J., Björklund A. (2013). TFEB-mediated autophagy rescues midbrain dopamine neurons from α-synuclein toxicity. Proc. Natl. Acad. Sci. USA.

[166] Phillips M.J., Voeltz G.K. (2016). Structure and function of ER membrane contact sites with other organelles. Nat. Rev. Mol. Cell Biol..

[167] Calì T., Ottolini D., Negro A., Brini M. (2012). α-Synuclein controls mitochondrial calcium homeostasis by enhancing endoplasmic reticulum-mitochondria interactions. J. Biol. Chem..

[168] Guardia-Laguarta C., Area-Gomez E., Rüb C., Liu Y., Magrané J., Becker D., Voos W., Schon E.A., Przedborski S. (2014). α-Synuclein is localized to mitochondria-associated ER membranes. J. Neurosci..

[169] Vicario M., Cieri D., Brini M., Calì T. (2018). The Close Encounter Between Alpha-Synuclein and Mitochondria. Front. Neurosci..

[170] Braak H., Del Tredici K., Rüb U., de Vos R.A., Jansen Steur E.N., Braak E. (2003). Staging of brain pathology related to sporadic Parkinson’s disease. Neurobiol. Aging.

[171] Braak H., Ghebremedhin E., Rüb U., Bratzke H., Del Tredici K. (2004). Stages in the development of Parkinson’s disease-related pathology. Cell. Tissue Res..

[172] Killinger B.A., Kordower J.H. (2019). Spreading of alpha-synuclein-relevant or epiphenomenon?. J. Neurochem..

[173] Bernal-Conde L.D., Ramos-Acevedo R., Reyes-Hernández M.A., Balbuena-Olvera A.J., Morales-Moreno I.D., Argüero-Sánchez R., Schüle B., Guerra-Crespo M. (2019). Alpha-Synuclein Physiology and Pathology: A Perspective on Cellular Structures and Organelles. Front. Neurosci..

[174] Chu Y., Morfini G.A., Langhamer L.B., He Y., Brady S.T., Kordower J.H. (2012). Alterations in axonal transport motor proteins in sporadic and experimental Parkinson’s disease. Brain.

[175] Prots I., Veber V., Brey S., Campioni S., Buder K., Riek R., Böhm K.J., Winner B. (2013). α-Synuclein oligomers impair neuronal microtubule-kinesin interplay. J. Biol. Chem..

[176] Tilve S., Difato F., Chieregatti E. (2015). Cofilin 1 activation prevents the defects in axon elongation and guidance induced by extracellular alpha-synuclein. Sci. Rep..

[177] Minakaki G., Krainc D., Burbulla L.F. (2020). The Convergence of Alpha-Synuclein, Mitochondrial, and Lysosomal Pathways in Vulnerability of Midbrain Dopaminergic Neurons in Parkinson’s Disease. Front. Cell. Dev. Biol..

[178] Xu L., Pu J. (2016). Alpha-Synuclein in Parkinson’s Disease: From Pathogenetic Dysfunction to Potential Clinical Application. Parkinsons Dis..

[179] Thayanidhi N., Helm J.R., Nycz D.C., Bentley M., Liang Y., Hay J.C. (2010). Alpha-synuclein delays endoplasmic reticulum (ER)-to-Golgi transport in mammalian cells by antagonizing ER/Golgi SNAREs. Mol. Biol. Cell.

[180] Ulusoy A., Decressac M., Kirik D., Björklund A. (2010). Viral vector-mediated overexpression of α-synuclein as a progressive model of Parkinson’s disease. Prog. Brain Res..

[181] Volpicelli-Daley L.A., Kirik D., Stoyka L.E., Standaert D.G., Harms A.S. (2016). How can rAAV-α-synuclein and the fibril α-synuclein models advance our understanding of Parkinson’s disease?. J. Neurochem..

[182] Xu S., Chan P. (2015). Interaction between Neuromelanin and Alpha-Synuclein in Parkinson’s Disease. Biomolecules.

[183] Villa S., Lombardi A., Mangioni D., Bozzi G., Bandera A., Gori A., Raviglione M.C. (2020). The COVID-19 pandemic preparedness … or lack thereof: From China to Italy. Glob. Health Med..

[184] Frigerio R., Fujishiro H., Ahn T.B., Josephs K.A., Maraganore D.M., DelleDonne A., Parisi J.E., Klos K.J., Boeve B.F., Dickson D.W. (2011). Incidental Lewy body disease: Do some cases represent a preclinical stage of dementia with Lewy bodies?. Neurobiol. Aging.

[185] Auluck P.K., Chan H.Y., Trojanowski J.Q., Lee V.M., Bonini N.M. (2002). Chaperone suppression of alpha-synuclein toxicity in a Drosophila model for Parkinson’s disease. Science.

[186] Ihara M., Yamasaki N., Hagiwara A., Tanigaki A., Kitano A., Hikawa R., Tomimoto H., Noda M., Takanashi M., Mori H. (2007). Sept4, a component of presynaptic scaffold and Lewy bodies, is required for the suppression of alpha-synuclein neurotoxicity. Neuron.

[187] Outeiro T.F., Kontopoulos E., Altmann S.M., Kufareva I., Strathearn K.E., Amore A.M., Volk C.B., Maxwell M.M., Rochet J.C., McLean P.J. (2007). Sirtuin 2 inhibitors rescue alpha-synuclein-mediated toxicity in models of Parkinson’s disease. Science.

[188] Li W., Lesuisse C., Xu Y., Troncoso J.C., Price D.L., Lee M.K. (2004). Stabilization of alpha-synuclein protein with aging and familial parkinson’s disease-linked A53T mutation. J. Neurosci..

[189] Bender A., Krishnan K.J., Morris C.M., Taylor G.A., Reeve A.K., Perry R.H., Jaros E., Hersheson J.S., Betts J., Klopstock T. (2006). High levels of mitochondrial DNA deletions in substantia nigra neurons in aging and Parkinson disease. Nat. Genet..

[190] Vilchez D., Saez I., Dillin A. (2014). The role of protein clearance mechanisms in organismal ageing and age-related diseases. Nat. Commun..

[191] Wong Y.C., Holzbaur E.L. (2015). Autophagosome dynamics in neurodegeneration at a glance. J. Cell Sci..

[192] Finkel T., Holbrook N.J. (2000). Oxidants, oxidative stress and the biology of ageing. Nature.

[193] Zecca L., Casella L., Albertini A., Bellei C., Zucca F.A., Engelen M., Zadlo A., Szewczyk G., Zareba M., Sarna T. (2008). Neuromelanin can protect against iron-mediated oxidative damage in system modeling iron overload of brain aging and Parkinson’s disease. J. Neurochem..

[194] Lee H.J., Suk J.E., Patrick C., Bae E.J., Cho J.H., Rho S., Hwang D., Masliah E., Lee S.J. (2010). Direct transfer of alpha-synuclein from neuron to astroglia causes inflammatory responses in synucleinopathies. J. Biol. Chem..

[195] Woerman A.L., Watts J.C., Aoyagi A., Giles K., Middleton L.T., Prusiner S.B. (2018). α-Synuclein: Multiple System Atrophy Prions. Cold Spring Harb. Perspect. Med..

[196] Ubhi K., Low P., Masliah E. (2011). Multiple system atrophy: A clinical and neuropathological perspective. Trends Neurosci..

[197] Ouchi Y., Yoshikawa E., Sekine Y., Futatsubashi M., Kanno T., Ogusu T., Torizuka T. (2005). Microglial activation and dopamine terminal loss in early Parkinson’s disease. Ann. Neurol..

[198] Gao H.M., Kotzbauer P.T., Uryu K., Leight S., Trojanowski J.Q., Lee V.M. (2008). Neuroinflammation and oxidation/nitration of alpha-synuclein linked to dopaminergic neurodegeneration. J. Neurosci..

[199] Harms A.S., Cao S., Rowse A.L., Thome A.D., Li X., Mangieri L.R., Cron R.Q., Shacka J.J., Raman C., Standaert D.G. (2013). MHCII is required for α-synuclein-induced activation of microglia, CD4 T cell proliferation, and dopaminergic neurodegeneration. J. Neurosci..

[200] Daniele S.G., Béraud D., Davenport C., Cheng K., Yin H., Maguire-Zeiss K.A. (2015). Activation of MyD88-dependent TLR1/2 signaling by misfolded α-synuclein, a protein linked to neurodegenerative disorders. Sci. Signal..

[201] Kim C., Ho D.H., Suk J.E., You S., Michael S., Kang J., Joong Lee S., Masliah E., Hwang D., Lee H.J. (2013). Neuron-released oligomeric α-synuclein is an endogenous agonist of TLR2 for paracrine activation of microglia. Nat. Commun..

[202] Wang S., Chu C.H., Stewart T., Ginghina C., Wang Y., Nie H., Guo M., Wilson B., Hong J.S., Zhang J. (2015). α-Synuclein, a chemoattractant, directs microglial migration via H2O2-dependent Lyn phosphorylation. Proc. Natl. Acad. Sci. USA.

[203] Thome A.D., Harms A.S., Volpicelli-Daley L.A., Standaert D.G. (2016). microRNA-155 Regulates Alpha-Synuclein-Induced Inflammatory Responses in Models of Parkinson Disease. J. Neurosci..

[204] Ejlerskov P., Hultberg J.G., Wang J., Carlsson R., Ambjørn M., Kuss M., Liu Y., Porcu G., Kolkova K., Friis Rundsten C. (2015). Lack of Neuronal IFN-β-IFNAR Causes Lewy Body- and Parkinson’s Disease-like Dementia. Cell.

[205] Hamza T.H., Zabetian C.P., Tenesa A., Laederach A., Montimurro J., Yearout D., Kay D.M., Doheny K.F., Paschall J., Pugh E. (2010). Common genetic variation in the HLA region is associated with late-onset sporadic Parkinson’s disease. Nat. Genet..

[206] Larson M.E., Sherman M.A., Greimel S., Kuskowski M., Schneider J.A., Bennett D.A., Lesné S.E. (2012). Soluble α-synuclein is a novel modulator of Alzheimer’s disease pathophysiology. J. Neurosci..

[207] Masliah E., Rockenstein E., Veinbergs I., Sagara Y., Mallory M., Hashimoto M., Mucke L. (2001). beta-amyloid peptides enhance alpha-synuclein accumulation and neuronal deficits in a transgenic mouse model linking Alzheimer’s disease and Parkinson’s disease. Proc. Natl. Acad. Sci. USA.

[208] Yoshimoto M., Iwai A., Kang D., Otero D.A., Xia Y., Saitoh T. (1995). NACP, the precursor protein of the non-amyloid beta/A4 protein (A beta) component of Alzheimer disease amyloid, binds A beta and stimulates A beta aggregation. Proc. Natl. Acad. Sci. USA.

[209] Bachhuber T., Katzmarski N., McCarter J.F., Loreth D., Tahirovic S., Kamp F., Abou-Ajram C., Nuscher B., Serrano-Pozo A., Müller A. (2015). Inhibition of amyloid-β plaque formation by α-synuclein. Nat. Med..

[210] Zhang X., Gao F., Wang D., Li C., Fu Y., He W., Zhang J. (2018). Tau Pathology in Parkinson’s Disease. Front. Neurol..

[211] Arima K., Hirai S., Sunohara N., Aoto K., Izumiyama Y., Uéda K., Ikeda K., Kawai M. (1999). Cellular co-localization of phosphorylated tau- and NACP/alpha-synuclein-epitopes in lewy bodies in sporadic Parkinson’s disease and in dementia with Lewy bodies. Brain Res..

[212] Haggerty T., Credle J., Rodriguez O., Wills J., Oaks A.W., Masliah E., Sidhu A. (2011). Hyperphosphorylated Tau in an α-synuclein-overexpressing transgenic model of Parkinson’s disease. Eur. J. Neurosci..

[213] Jaworski T., Kügler S., Van Leuven F. (2010). Modeling of tau-mediated synaptic and neuronal degeneration in Alzheimer’s disease. Int. J. Alzheimers Dis..

